# Nonvolatile Memristive Materials and Physical Modeling for In‐Memory and In‐Sensor Computing

**DOI:** 10.1002/smsc.202300139

**Published:** 2024-01-22

**Authors:** Shao-Xiang Go, Kian-Guan Lim, Tae-Hoon Lee, Desmond K. Loke

**Affiliations:** ^1^ Department of Science, Mathematics and Technology Singapore University of Technology and Design Singapore 487372 Singapore; ^2^ Department of Engineering University of Cambridge Trumpington Street Cambridge CB2 1PZ UK; ^3^ School of Materials Science and Engineering Kyungpook National University Daegu 41566 Republic of Korea

**Keywords:** brain-inspired neuromorphic computing, in-memory computing, in-sensor computing, molecular dynamics simulations, nonvolatile memristive materials, physical unclonable functions

## Abstract

Separate memory and processing units are utilized in conventional von Neumann computational architectures. However, regarding the energy and the time, it is costly to shuffle data between the memory and the processing entity, and for data‐intensive applications associated with artificial intelligence, the demand is ever increasing. A paradigm shift in traditional architectures is required, and in‐memory computing is one of the non‐von‐Neumann computing strategies. By harnessing physical signatures of the memory, computing workloads are administered in the same memory element. For in‐memory computing, a wide range of memristive material (MM) systems have been examined. Moreover, developing computing schemes that perform in the same sensory network and that minimize the data shuffle between the processing unit and the sensing element is a requirement, to process large volumes of data efficiently and decrease the energy consumption. In this review, an overview of the switching character and system signature harnessed in three archetypal MM systems is rendered, along with an integrated application survey for developing in‐sensor and in‐memory computing, viz., brain‐inspired or analogue computing, physical unclonable functions, and random number generators. The recent progress in theoretical studies that reveal the structural origin of the fast‐switching ability of the MM system is further summarized.

## Graphical abstract

This review explores the utilization of memristive materials (MMs) as digital building blocks for in‐memory and in‐sensor computing. Switching mechanisms, material layer structures, MM operations, material system characteristics, theoretical studies, applications, and challenges and prospects for in‐sensor and in‐memory computations, for example, brain‐inspired neuromorphic or analogue computing, physical unclonable functions, and random number generators, are discussed.

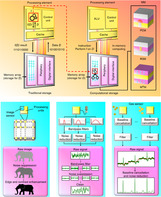

## 1 Introduction

Modern computation systems are constructed on the foundation of a von Neumann computing architecture in which data are transferred to a processing unit.^[^ ^1^, ^2^, ^3^ ^]^ Substantial costs in the energy and the latency, i.e., the delay between an instruction to transfer data and the same data being transferred, are incurred when a large amount of data are shuttled between the memory and the processing unit for performing different computing workloads. For many applications, e.g., vital artificial intelligence (AI)‐type tasks, the latency related to retrieving data from memory units is a major performance roadblock (researchers have called it a bottleneck). Between the time utilized for accessing data in the memory and the processing element, there is an increased difference, and this phenomenon is described as a memory wall. Since computational systems are energy constrained owing to the increase in the number of edge‐computing devices and cooling limitations, the energy cost of moving data is also a substantial challenge. The latency cost of multiplying two numbers in processing units is smaller compared to that of retrieving the numbers from the memory for traditional complementary metal–oxide–semiconductor (CMOS) technologies.^[^ ^4^, ^5^, ^6^ ^]^ The difficulty of avoiding data movement exists for conventional strategies, including the utility of application‐specific processors that are customized for targeted applications or many processors connected in parallel, viz., graphic processing units. Thus, new computing architectures in which the memory and the processing entity are more co‐located are required. Inserting a monolithic‐compute unit nearer to monolithic‐memory units physically is one of the concepts proposed.^[^ ^7^, ^8^, ^9^ ^]^ The recent enhancements in hardware stacking technologies and commercialization of advanced memory types including the high‐bandwidth memory and hybrid memory cubes have benefitted the idea, which is termed as near‐memory computing, substantially.^[^ ^10^, ^11^, ^12^ ^]^ The 3D monolithic integration was utilized further for attaining a smaller hardware size and a denser connectivity between the memory and processing units.^[^ ^13^, ^14^, ^15^ ^]^ However, a separation between the memory and the compute unit still exists physically for conventional schemes that target to minimize the distance and the time for memory retrieval.

**Figure 1:**
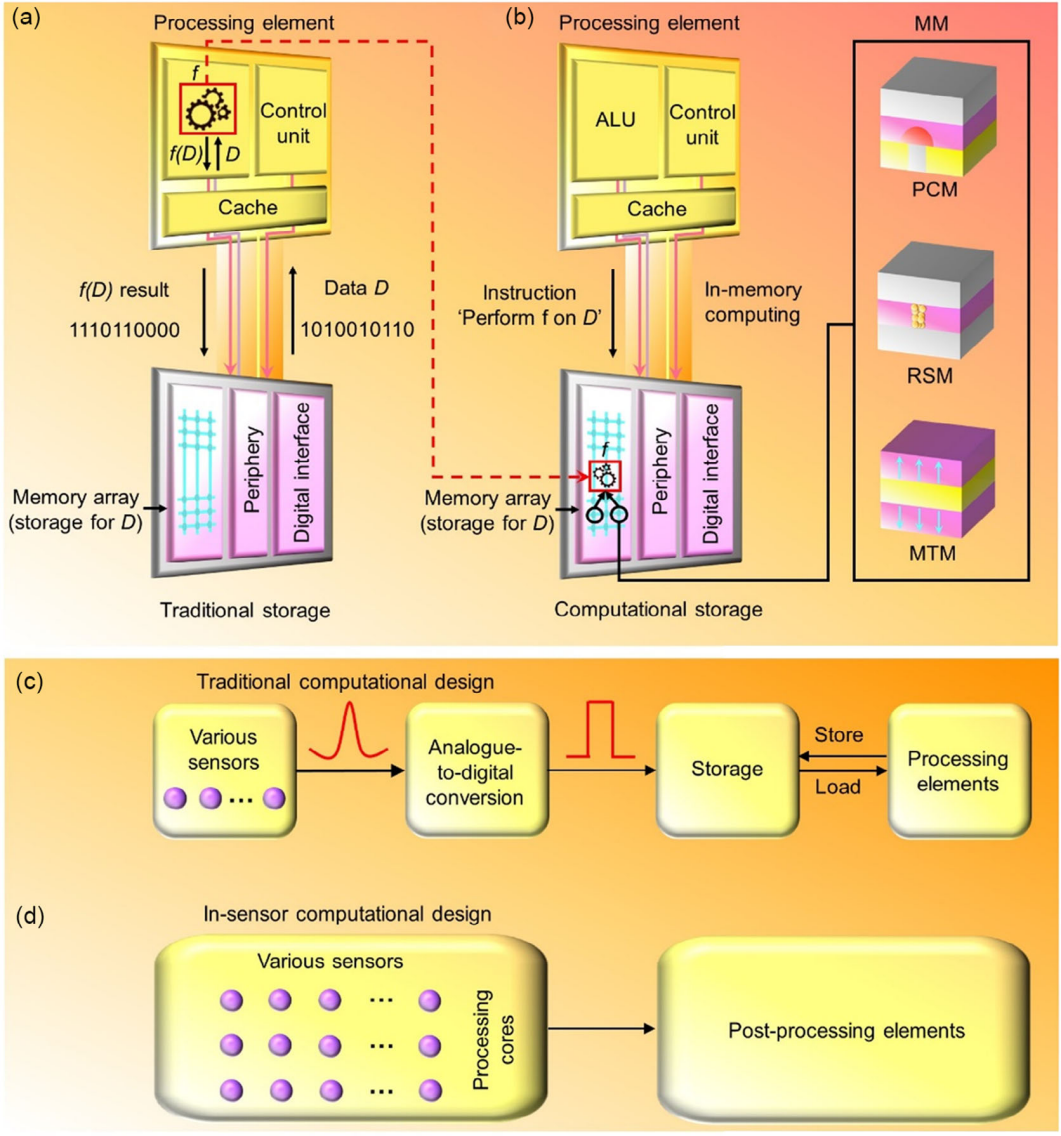
In‐memory computing and in‐sensor computation using MM systems. a) In a traditional computational platform, the data *D* are transferred to a processing element, when a function *f* is implemented on the *D*, resulting in substantial costs in power and time. b) With reference to the in‐memory computation, by harnessing physical characters of the memory hardware, the *f*(*D*) is administered in the same computational storage entity, therefore avoiding the requirement to transfer the *D* to the processing element. The MM technologies including phase‐change memory (PCM), resistive switching memory (RSM), and magnetic tunnelling memory (MTM) can operate as units of the computational‐storage element. c) Traditional sensory computational design. The analog outputs from different sensors are altered to digital signals, which are retained in the storage unit. The processing elements access the data from the storage and subsequently transfer the output signal back to the storage element for long‐term retention. d) The in‐sensor computational design. The processing operations are included in distinct sensors for front‐end processing. To avoid data transfer between sensors and processors, the sensor can cooperate to implement information aggregation and compression, and data processing.

A different strategy wherein computing workloads are implemented in the same memory entity is described as in‐memory computing (**Figure** **1**a,b).^[^ ^16^, ^17^, ^18^ ^]^ This is attained by harnessing the physical character of material systems, device designs, array‐type configurations, peripheral circuitries, and memory controllers.^[^ ^5^, ^19^, ^20^ ^]^ In‐memory computing describes a computing workload that is achieved in a memory unit. Moreover, in‐memory computing is a promising candidate for enhancing the time complexity, viz., the amount of computational time utilized to run an algorithm, of computing workloads.^[^ ^21^, ^22^, ^23^ ^]^ A high degree of parallelism enabled by a large ensemble of memory units that administer a computing task results in the improved time complexity. Furthermore, the time complexity decreases with an increase in the degree of connectivity between memory units. Thus, a substantial improvement in the computing efficiency is achieved when the boundary between the memory and the processing module becomes negligible, which mimics an energy efficient human brain where the processing and memory elements are interlinked.^[^ ^24^, ^25^, ^26^ ^]^

Memristive materials (we term them MMs) are promising candidates for achieving in‐memory computing.^[^ ^27^, ^28^, ^29^, ^30^, ^31^ ^]^ Upon the application of an external electrical stimulus, the MM system discloses programmable conductance states.^[^ ^32^, ^33^, ^34^ ^]^ The prototypical MM operations, enabled by the phase‐change, i.e., thermally induced crystalline–amorphous transitions, tunnel magnetoresistance, viz., spin‐dependent tunnel conductance, and electrochemical reaction, e.g., redox and ion migration, are based on the switching of a dielectric layer in a two‐terminal metal–dielectric–metal configuration.^[^ ^35^, ^36^, ^37^ ^]^ The MM systems, which have a small footprint, low programming energy, high reliability, and short switching time, exhibit a marked contrast in the electrical conductance as a result of the dependence of conductance states on the history of electrical stimuli.^[^ ^38^, ^39^, ^40^ ^]^ Furthermore, through the utilization of physical processes to perform complex signal alterations, the data can be processed in the MM system inherently for enhancing both the energy efficiency and the area efficacy of many applications, such as hardware security, and neuromorphic and analog computing.^[^ ^41^, ^42^, ^43^ ^]^

**Figure 2:**
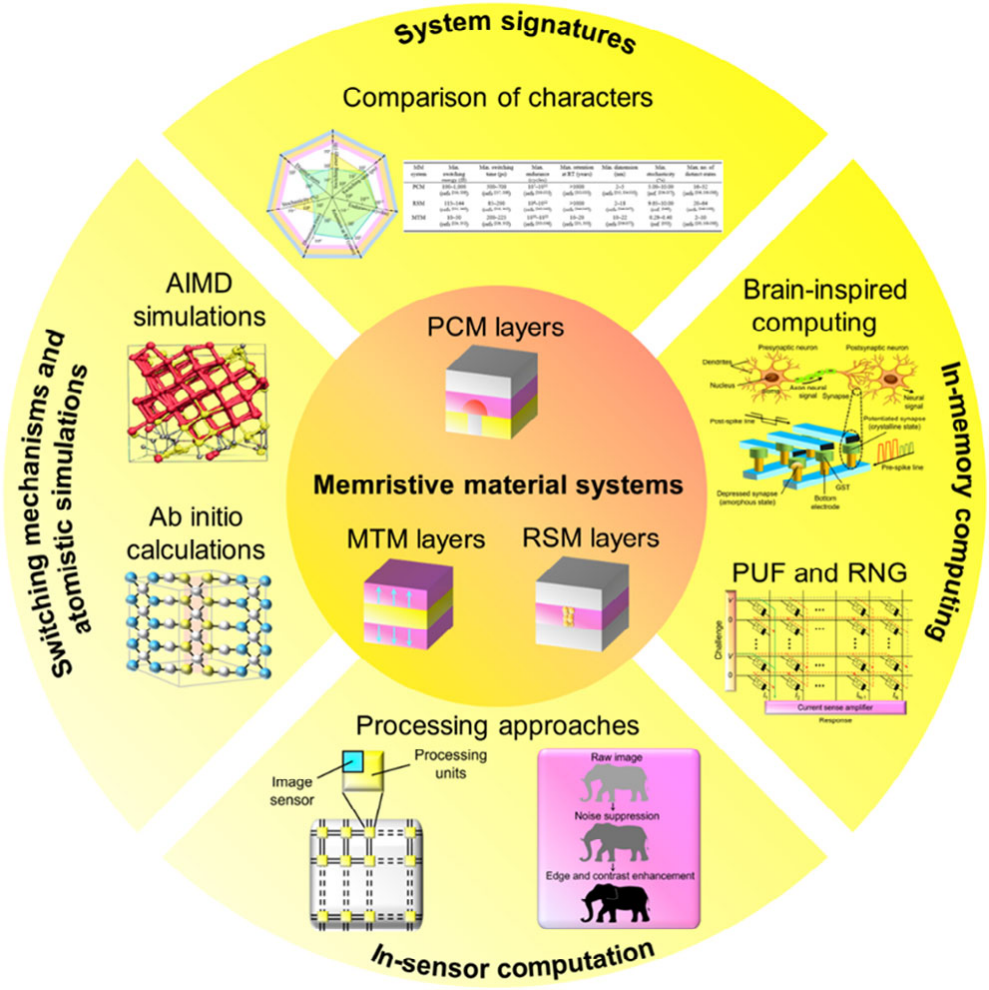
Memristive material development. Various types of memrisitive material systems, i.e., phase‐change memory layers, resistive switching memory layers, and magnetic tunneling memory layers, are disclosed. This survey presents four key modes of research domains that utilizes the memristive material system.

The analog sensory data are altered to digital signals through an analog‐to‐digital conversion, and subsequently stored in the memory, in traditional computing architectures (Figure 1c,d). Finally, the data are moved from the memory unit to processing units. However, a low‐energy efficiency, as well as long latency, results from the traditional data conversion and transmission strategy. Nevertheless, various connected sensors or single self‐adaptive sensor types process the sensory data directly, which integrate computing operations and sensing functions, in an in‐sensor computation architecture.^[^ ^44^, ^45^, ^46^, ^47^ ^]^ In this review, we provide an overview of the switching signature and system character utilized in three typical MM systems, along with a combined application survey for developing in‐memory computing, as well as in‐sensor computation (**Figure** **2**). We also disclose an outlook on the challenge and opportunities. Furthermore, we summarize theoretical studies that reveal the physical origin of the rapid‐switching ability of the MM system.

## 2 MM Switching Mechanisms and Atomistic Simulations

### 2.1 PCM‐Layer Switching Character

**Figure 3:**
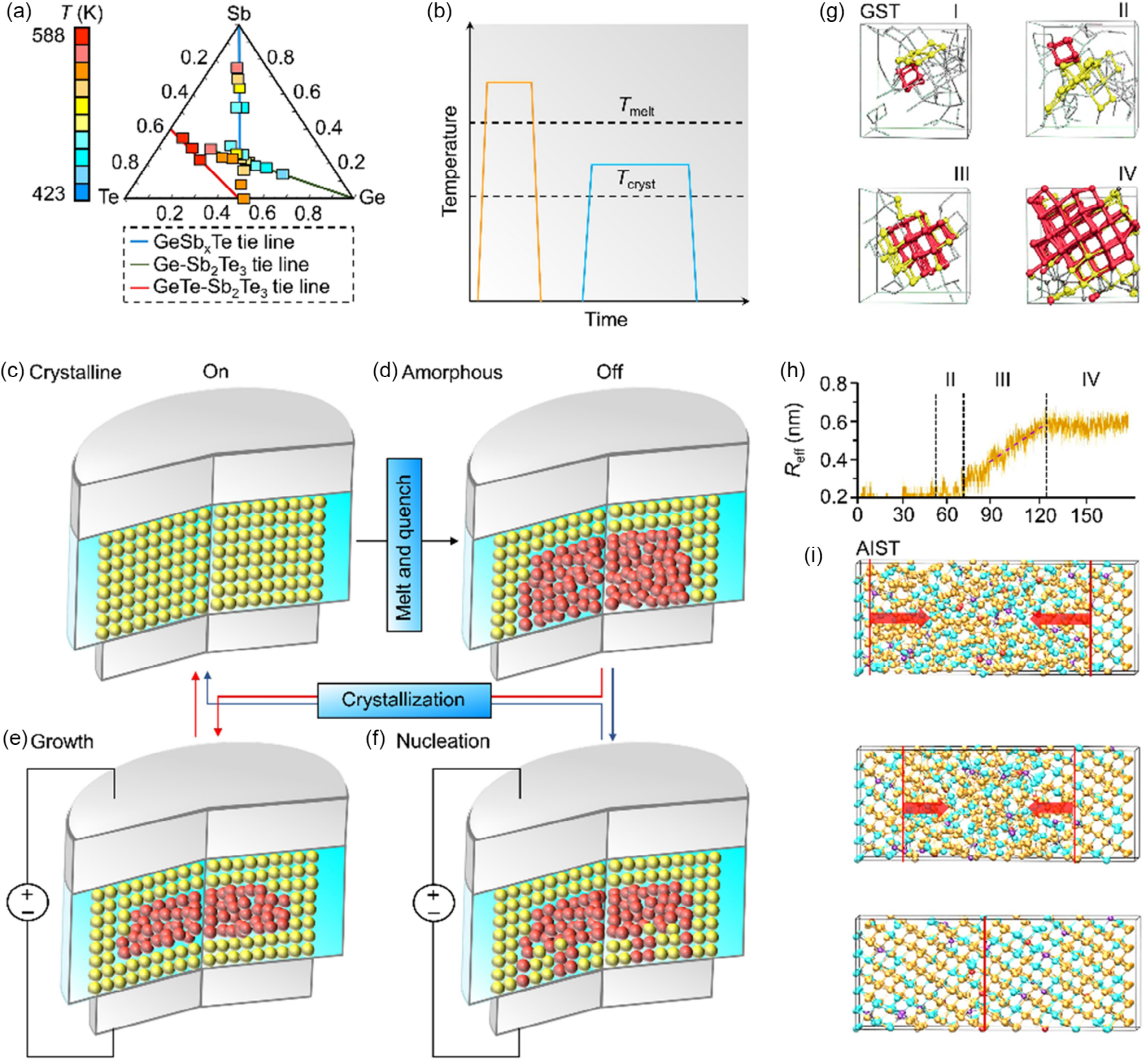
PCM layers and AIMD simulations. a) The germanium–antimony–telluride ternary phase diagram revealing the variation of the crystallization temperature for different PCM compositions. Adapted with permission.^[^ ^509^ ^]^ Copyright 2011, Institute of Electrical and Electronics Engineers. b) Temperature variations result in reversible phase transitions between the crystallized phase and the disordered amorphous phase. The *T*_cryst_ represents the crystallization point, while the *T*_melt_ denotes the melting temperature. c,d) Schematic representations of the crystallized state (c) and the glassy amorphous state (d) of the PCM element. The heating above the *T*_melt_ to melt and subsequently quench the PCM volume rapidly results in switching the crystallized phase into the disordered‐amorphous phase. On the other hand, the heating above the *T*_cryst_ for an intermediate time to crystallize the PCM element leads to switching the amorphous phase into the ordered crystallized phase. e,f) Schematic diagrams of the (e) growth‐dominated and (f) nucleation‐dominated crystallization procedures. g) Snapshots of a GeSbTe model utilized in an ab initio molecular dynamics (AIMD) simulation at ≈600 K. g) Adapted with permission.^[^ ^132^ ^]^ Copyright 2011, American Physical Society. h) The progression of the effective radius (*R*_eff_) of crystallized clusters from a crystallization trajectory of the GeSbTe. Adapted with permission.^[^ ^132^ ^]^ Copyright 2011, American Physical Society. i) The snapshots of the crystal growth from crystallized–amorphous boundaries in the AgInSbTe at ≈590 K. In the interface growth procedure, negligible substantial crystallized clusters were generated. Adapted with permission.^[^ ^114^ ^]^ Copyright 2014, The Authors, published by Springer Nature.

Comprising traditional elements, viz., Ge, Te, and/or Sb, phase‐change memory (PCM) layers are driven by a reversible switching in an ensemble of glassy chalcogenide alloys in the Ge–Sb–Te ternary phase diagram (**Figure** **3**a).^[^ ^48^, ^49^ ^]^ The electrical conductivity of the PCM layer is small in the metastable‐amorphous phase, which discloses a short‐range order, whereas in the crystallized phase that reveals a long‐range order, the electrical conductivity is large.^[^ ^50^ ^]^ The creation of previous optical‐rewritable disks resulted from a marked optical contrast elucidated by the crystallized phase and the glassy amorphous phase.^[^ ^51^ ^]^ Understanding the large variation in macroscopic signatures of material phases as a result of small variations in atomic positions continues to be of interest to the research community.^[^ ^52^, ^53^ ^]^ Temperature alterations enabled by the passive thermal dissipation and Joule heating induce the electrical switching of PCM layers (Figure 3b). Heating the PCM layer above the melting temperature, i.e., ≈900 K, and subsequent rapid quenching of the undercooled liquid result in switching the crystallized phase into the disordered amorphous phase (Figure 3c,d). Facilitated by the nanosized melted volume and high thermal conductivity electrodes, the electrical heating energy peaks in the melting stage, and the passive cooling is adequate for the amorphization in sub‐nanosecond timescales.^[^ ^54^, ^55^, ^56^, ^57^, ^58^, ^59^ ^]^ Maintaining the temperature of the amorphous PCM layer above the crystallization point, i.e., ≈600 K, and over a specified period to induce the crystallization leads to the switching into the crystallized phase.^[^ ^60^, ^61^, ^62^, ^63^, ^64^ ^]^ Recent studies have disclosed rapid crystallizing materials, since the traditional crystallization time is on the twenty to a thousand nanosecond timescales, which limits the programming speed of the PCM layer.

#### 2.1.1 Nucleation and Growth Processes

The PCM layers are grouped into the nucleation‐dominated material or growth‐dominated materials based on thermodynamic factors, e.g., the free energy as a function of the interfacial area and the targeted nuclei radius, in the crystallization process.^[^ ^65^, ^66^ ^]^ A single crystallized phase results when the growth‐dominated PCM layer recrystallizes at the interface between the amorphous volume and the ordered crystallized volume (Figure 3e). The Ag‐ and In‐doped SbTe, viz., the Ag_4_In_3_Sb_67_Te_26_ (AIST), is an archetype. On the other hand, the nucleation‐dominated PCM layer discloses the critical‐nuclei formation and is observed in a majority of PCM layers. The nucleation and growth are rapid for the nucleation‐dominated PCM layer including the Ge_2_Sb_2_Te_5_ (GST). A polycrystalline phase with a varied grain orientation results from the nuclei formation and subsequent fast grain growth (Figure 3f).^[^ ^67^, ^68^ ^]^

The GST and AIST crystallization processes differ owing to differences in nucleation rates, which are influenced by thermal fluctuations. The interfacial energy and driving force influence the height of the free energy barrier. According to classical nucleation theory, when atoms are incorporated into a subcritical nucleus, the free energy increases, subsequently decreasing until the nucleus reaches a critical size. The size of the critical nucleus diminishes as the interfacial energy decreases, or the driving force increases. The critical size alters with temperature, with an average interfacial energy of ≈35 meV atom^−1^ at 800 K.^[^ ^69^, ^70^ ^]^ In accordance with classical nucleation theory, a temperature induces a peak nucleation rate owing to two aspects: decreasing atomic jump rate and increasing driving force.^[^ ^71^ ^]^ However, owing to nonequilibrium dynamics and nonisothermal instances in PCM elements, this estimation is inadequate for rapid nucleation events observed in traditional PCM layers, rendering it difficult to adequately represent the nucleation event. The GST layers require several nanoseconds to incubate at high temperatures, whereas over 20 s are utilized for the case of the AIST layer.^[^ ^72^, ^73^ ^]^ Both layers are 30 nm thick and capped with films that inhibit boundary‐assisted crystal growth and minimize oxidation. The AIST and GST nucleation rates vary, according to structural characterization studies and ab initio simulations.

Crystallization in AIST is growth dominated and GeSbTe is typically nucleation dominated, but the crystallization process may be predominantly induced by crystal growth in nanosized PCM layers.^[^ ^74^, ^75^ ^]^ The effect of nucleation may be less vital in the nanosized PCM element since the steady‐state rate is temporally dependent on the nucleation rate.^[^ ^76^, ^77^, ^78^ ^]^ If the growth rate is sufficiently high for substantial growth at the crystallized–amorphous interface to occur prior to the incubation period for nucleation, crystallization is growth driven in nucleation‐dominated PCM layers, specifically in nanosized PCM elements with a large ratio of amorphous–crystallized interface area to amorphous region volume.^[^ ^79^, ^80^ ^]^ Owing to the existence of multiple quenched‐in crystallized nuclei in the melt‐quenched amorphous phase of GST, recent studies reveal that crystal growth is more prevalent than nucleation in PCM elements.^[^ ^81^, ^82^ ^]^ This indicates that, if new nuclei appear during crystallization, the growth of existing quench‐in nuclei may dominate the crystallization time.

A widely known model describes the temperature dependence of crystal growth, which is denoted by

(1) $$v_{g}\left(T\right)=\frac{4r_{a}\cdot k_{B}T}{3\pi \lambda^{2}R_{h}}\cdot \frac{1}{\eta \left(T\right)}\cdot \left(1-\text{exp}\left(-\frac{\Delta G\left(T\right)}{k_{B}T}\right)\right)$$

where $r_{a}$ represents the atomic radius, *λ* describes the diffusional jump distance, $R_{h}$ is the hydrodynamic radius, $k_{B}$ depicts the Boltzmann constant, and *T* is the temperature.^[^ ^76^, ^83^ ^]^ The Gibbs energy difference, i.e., ΔG(T), influences the energy difference between the crystallized phase and the liquid phase during crystallization. The ΔG(T) is typically more than zero for $T<T_{\text{melt}}$, equal to zero for $T=T_{\text{melt}}$, and less than zero for $T>T_{\text{melt}}$. Thermally induced atomic transport across the solid–liquid interface is represented by the expression in large round brackets. The Thomson–Spaepen estimation, utilized by recent works, is a frequently used equation for ΔG in PCM layers.^[^ ^83^, ^84^, ^85^ ^]^

#### 2.1.2 Progressive Crystallization

The binary switching, e.g., the set transition from the low conductance state to the high‐conductance state, has been harnessed for brain‐inspired neuromorphic and analog computing using PCM layers. By expanding to the multilevel‐transition realm, in which administering constant stimuli results in a partial, amendable change in the material conductance, a larger degree of freedom was achieved.^[^ ^86^, ^87^, ^88^ ^]^ For instance, an increased crystallized bit results in an increasing conductance in the PCM layer, wherein the set transition comprises a gradual crystallization of the amorphous volume. As the number of stimuli increases, the PCM layer changes from the amorphous state to the crystallized state. Moreover, recent studies have disclosed the crystallized–amorphous phase distribution in the PCM layer for an increased crystallization period and simulated‐temperature profile during programming operations.^[^ ^89^ ^]^ The thickness of the amorphous layer decreases with an increased time owing to the crystallization process, leading to an increasing material conductance and a decreased threshold voltage *V*_T_.

#### 2.1.3 Conductance Drift

The conductance drift in traditional amorphous PCM layers at room temperature impedes long‐term ultracompact multilevel data storage.^[^ ^90^, ^91^ ^]^ The electrical conductance consistently and gradually decreases, suggesting a structural transition or relaxation to an ultrastable or ideal glassy state.^[^ ^92^, ^93^, ^94^ ^]^ The destruction of tetrahedrally coordinated Ge and homopolar bonds and strengthening of bond distortions are connected to the aging process in Ge‐rich PCM layers.^[^ ^95^, ^96^ ^]^ The aging occurs in the Ge‐free PCM layer as well.^[^ ^95^, ^97^ ^]^ At 100 K, pristine amorphous Sb exhibits a conductance drift phenomenon with a drift exponent comparable to GST at ambient temperature.^[^ ^98^, ^99^ ^]^ When in contact with dielectric materials or metals, however, amorphous PCM layers exhibit low drift exponents.^[^ ^100^, ^101^ ^]^ Moreover, the time dependency of PCM layer's low‐field conductance under constant room temperature is characterized by

(2) $$G(t)=G\left(t_{0}\right){\left(\frac{t}{t_{0}}\right)}^{-v}$$

where $G\left(t_{0}\right)$ describes the conductance measured at time $t_{0}$ and *v* is the drift exponent, which discloses a value of ≈0.1 for the reset state. The conductance drift is induced by amorphous PCM layers, whereas in the case of the set state, small drift exponents are exhibited, viz., 0.05. Conductance drift is a challenge for multilevel storage since the conductance drift phenomenon restricts the number of levels that can be successfully retrieved and stored in a traditional memory element.^[^ ^102^, ^103^ ^]^ Conductance drift exhibits consequences in conventional non‐von‐Neumann computing applications as well.^[^ ^104^ ^]^

### 2.2 Atomistic Modeling of PCM Layers

Amorphous PCM layers are difficult to investigate owing to the complicated chemical composition and disordered configuration. Ab initio molecular dynamics (AIMD) simulations based on density functional theory (DFT) have been vital in computing interatomic forces with quantum mechanics precision, rendering AIMD simulations widely utilized for examining the material signature and structure.^[^ ^72^, ^105^, ^106^, ^107^, ^108^ ^]^ The AIMD simulations can be used to investigate models including hundreds of atoms in a few hundreds of picoseconds of simulation time, with specified optimization using improved DFT programs for PCM layers.^[^ ^109^, ^110^, ^111^ ^]^ The simulations may be applied to determine structural factors, as well as vibrational and optical spectra, which can then be compared with experiments, e.g., Raman spectroscopy, photothermal deflection spectroscopy, transmission electron microscopy (TEM), X‐Ray diffraction and absorption, and other experiments. Reverse Monte Carlo analysis is also utilized to generate amorphous models or enhance AMID findings that are restricted by experimental data.

#### 2.2.1 Structural Character

The GeSbTe crystallizes into a cubic rocksalt phase with a Te sublattice and a randomly populated other sublattice with 40% Ge, 40% Sb, and 20% atomic vacancies after nanosecond switching.^[^ ^112^, ^113^ ^]^ Recrystallized AIST generates an A7 configuration with a statistical element distribution, resulting in disordered crystallized states with defective or distorted octahedral coordination.^[^ ^114^, ^115^ ^]^ The amorphous configuration of conventional GeSbTe and GeTe types has been discussed. Recent studies reveal that the majority of Ge atoms are tetrahedrally coordinated, which leads to the umbrella flip concept for rapid crystallization.^[^ ^51^, ^116^ ^]^ However, AIMD simulations demonstrated that the majority of Ge atoms were in defective octahedral coordination.^[^ ^105^, ^117^ ^]^ The tetrahedral Ge units are locally stabilized by quenched‐in homopolar Ge—Ge bonds and are projected to vanish during structural relaxation.^[^ ^95^, ^118^, ^119^ ^]^ This model agrees well with observations of X‐ray absorption near‐edge and TEM data paired with local reverse Monte Carlo simulations.^[^ ^120^, ^121^ ^]^ The Ge atoms are present in an octahedral coordination in amorphous GST, although with a higher degree of bond distortion compared to the crystallized equivalents.^[^ ^122^, ^123^ ^]^ The primitive ring scheme is utilized to examine the crystallization process of rocksalt GeSbTe and AIST.^[^ ^115^, ^124^ ^]^ Fourfold primitive rings are the most prevalent structural units in amorphous GeSbTe, with over 80% exhibiting the configuration ABAB.^[^ ^106^, ^124^ ^]^ These fourfold rings are the smallest structural entities in rocksalt GeSbTe and are important structures for rapid nucleation.^[^ ^124^, ^125^ ^]^ Amorphous AIST, on the other hand, exhibits a high distribution of primitive rings ranging from threefold to sevenfold, with fivefold primitive rings being the most typical. These structural units vary from the crystallized AIST's local octahedral environment, resulting in a decreased nucleation rate.^[^ ^115^, ^126^ ^]^

#### 2.2.2 Basis of Nucleation

The postulated connection between local structural motifs in the amorphous phase at room temperature and nucleation rate at increased temperatures is investigated using direct dynamical simulations of the crystallization process of PCMs. Due to the capacity to depict complicated and swiftly changing chemical combinations and quantum mechanical precision, AIMD simulations are widely utilized. AIMD simulations may be used at an appropriate computing cost on small timeframes and length scales associated with the crystallization process in ultrasmall PCM elements. The first AIMD crystallization simulation of GeSbTe was fulfilled using a 72 atom configuration during liquid‐state quenching, disclosing GeSbTe's rapid crystallization propensity.^[^ ^127^ ^]^

The GeSbTe nucleates quickly, but traditional GeSbTe types are unable to rival with rapid static random‐access memory (SRAM) and dynamic random‐access memory (DRAM) that necessitates sub‐nanosecond switching times. A preprogramming or priming approach was generated to boost crystallization time at high temperatures without influencing long‐term data retention.^[^ ^72^, ^128^ ^]^ This strategy includes administering a constant low bias or voltage to a 30 nm‐thick pore‐based PCM element, resulting in a set time of 500 ps. AIMD simulations elucidate that the constant voltage‐based prestructural ordering or incubation methodology induces preseeding of nuclei within the amorphous matrix, enabling the PCM layer to switch to the crystallized state through crystal growth from numerous nuclei.

Recent studies demonstrated that Sc_0.2_Sb_2_Te_3_ is a promising material system for PCM applications, with a conductance contrast of two orders of magnitude between the amorphous phase and the rocksalt phase.^[^ ^129^ ^]^ The amorphous structure of Sc_0.2_Sb_2_Te_3_ created by AIMD simulations is composed of fourfold primitive rings, with all Sc atoms being included in the ABAB ring. During AIMD simulations at 600 K, the strong Sc—Te bonds and cubes stayed intact for 50 ps, demonstrating the high stability against thermal fluctuations and the potential of generating large seeds. Crystallized seeds containing ≈50 atoms may adapt to thermal variations in Sc_0.2_Sb_2_Te_3_ at 600 K, enabling rapid crystal formation. An ≈400 atom Sc_0.2_Sb_2_Te_3_ configuration demonstrated rapid crystallization in 600 ps. Enhanced crystallization was exhibited by Sc_0.2_Sb_2_Te_3_‐based PCM elements, with sub‐nanosecond programming times. The presence of a large population of dynamically resilient crystallized entities is vital for rapid PCM crystallization.

#### 2.2.3 Crystal Growth at Microscopic Scale

A rapid crystal growth speed leads to a fast crystallization in PCM layers. For example, although crystallized species, i.e., tetrahedral SiO_4_ archetypes, are robust and abundant in the amorphous volume,^[^ ^130^, ^131^ ^]^ the conventional liquid SiO_2_ discloses an upper bound of the crystal growth speed of ≈10^−9^ m s^−1^. However, the associated set time attained for the traditional SiO_2_ layer is on several‐second timescales, which is lengthy for data storage uses and in‐memory computing applications. Thus, for the case of the GST, since the set process is achieved below a nanosecond, the GST layer reveals a rapid crystal growth speed. Moreover, a fast crystal growth from nuclei was attained in an approximately two hundred atom model of PCM layers at 600 K, in atomistic simulations of the crystallization of the GST (Figure 3g,h).^[^ ^132^ ^]^ A growth rate of ≈5 m s^−1^ was obtained by estimating the variation in the effective radius with time, which was above that of experimental data at ≈600 K.^[^ ^66^, ^133^ ^]^ To enhance the model accuracy, larger models comprising four hundred to nine hundred atoms were utilized.^[^ ^134^ ^]^ To decrease the computational cost and facilitate the critical nucleus formation, an improved sampling methodology was harnessed.^[^ ^135^ ^]^ To calculate the growth rate, a bond‐order correlation parameter that differentiates atoms in an amorphous configuration from those in a crystallized framework was used.^[^ ^136^ ^]^ A growth rate of ≈1.0 m s^−1^ at 600 K was obtained in the four hundred atom model, and for the case of nine hundred atom models, the growth rate decreases to 0.5 m s^−1^, which agrees well with experimental results.^[^ ^110^, ^111^, ^137^ ^]^

The configuration of the amorphous–crystallized interface was facilitated to examine the crystal growth process in the PCM layer quantitatively. For instance, researchers have investigated the growth‐dominated material AIST with a growth direction in the [0001] crystal orientation.^[^ ^114^ ^]^ An amorphous model comprising two uniform amorphous–crystallized interfaces (the interface was created due to periodic‐boundary conditions) was generated by inserting two side‐by‐side crystallized layers in a melt‐quench process (Figure 3i). Approximately eight hundred atoms and the stoichiometry AIST were utilized for the amorphous model. Negligible large‐crystallized seeds were disclosed in the disordered layer upon the heating at 600 K and the crystallization occurred rapidly through the model interface, indicating a boundary‐enhanced growth process. A growth rate of ≈7 m s^−1^ along the [0001] growth direction was attained by computing the variation in the ratio of atoms in a crystallized configuration with time. The value agrees well with experimental data at ≈600 K.^[^ ^83^ ^]^ On the other hand, a corresponding ensemble of simulations performed for the GST along the [111] orientation of the cubic phase reveals a growth rate of ≈1 m s^−1^, which is consistent with experimental findings.^[^ ^137^ ^]^ As a result of the fast crystal‐growth rate of the GST, the GST‐memory element elucidates a growth‐dominated character if surrounding element layers facilitate the boundary‐enhanced crystal growth. A several nanometre‐thick amorphous GST that crystallizes through the boundary‐assisted crystal growth process is an example.^[^ ^138^ ^]^

The narrow interface, efficient atomic‐attachment process, i.e., the large driving force owing to the deep undercooling, and the high mobility in the PCM layer result in the rapid crystal growth of GST and AIST.^[^ ^95^ ^]^ For the GST, the computed bulk diffusivity was ≈1.5 × 10^−10^ m^2^ s^−1^ at 600 K, whereas the calculated bulk‐diffusivity value was 5.0 × 10^−10^ m^2^ s^−1^ at ≈600 K for the case of the AIST. The constant and rapid collision of atoms onto the crystallized boundary was indicated by the ≈0.5 nm‐thick narrow interface achieved in both GST and AIST, as disclosed in plots of the diffusivity along the growth direction and the bond‐order parameter. The probability that an atom collides at a crystallized site of the interface and attach to the crystal constantly is represented by the attachment coefficient. For GST and AIST, calculated attachment coefficients at 600 K were 0.2 and ≈0.4, respectively, which indicated that the attachment process was effective.^[^ ^110^ ^]^ Furthermore, the fundamental origin of the fast crystallization of PCM layers arises from the unique chemical bonding character of the GST, which leads to facile local, structural changes around constituent atoms, i.e., including atoms at the crystallized–amorphous interface.^[^ ^139^, ^140^, ^141^ ^]^

### 2.3 RSM Layer Switching Signature

In the filamentary conduction process, the resistive switching mechanism includes the growth and breakage of conductive filament in the resistive switching memory (RSM) layer. Cation‐dominated filament growth processes are distinguished from the anion‐dominated filament growth process. Under an electric field, charged ions or defects move and amass, linking the top electrode to the bottom electrode to create the conductive filament. The charged ions or defects migrate and fracture the conductive filament in the presence of a reverse field, resulting in the generation of nonvolatile bipolar resistive switching. A prototypical bipolar resistive switching may be observed in a cation‐filament‐growth‐based RSM layer. The cation‐dominated filament growth processes utilize charged ions, viz., top electrode metal ions, whereas defects, e.g., oxygen vacancies, are required in the case of the anion‐dominated filament growth process. The unipolar resistive switching character in both conductive filament growth processes is understood as a conductive filament model based on the Joule heating effect. However, resistive‐switching signatures of unipolar RSM layers are independent of the administered voltage, but a voltage dependence is exhibited in the case of the resistive‐switching signature of the bipolar RSM layer. This character is conspicuous in NiO‐based RSM layers.^[^ ^142^, ^143^ ^]^ The traditional conductive filament formation and rupture display a variable signature, with the conductive filament not always dissolving fully throughout the reset process.^[^ ^144^, ^145^ ^]^ The conductive filament is generally created using set voltages smaller than the forming‐set voltage. Ultrathin conductive filament branches are created by traditional large electrode spacing, which has a specified influence on material performance.^[^ ^146^, ^147^ ^]^ Unbounded conductive filament growth induces uncertainty, while growth space is constrained when prototypical conductive filament elements are incorporated at a high density. This issue may be alleviated by assuring that the element size is small enough.

#### 2.3.1 Anion‐Based Switching

**Figure 4:**
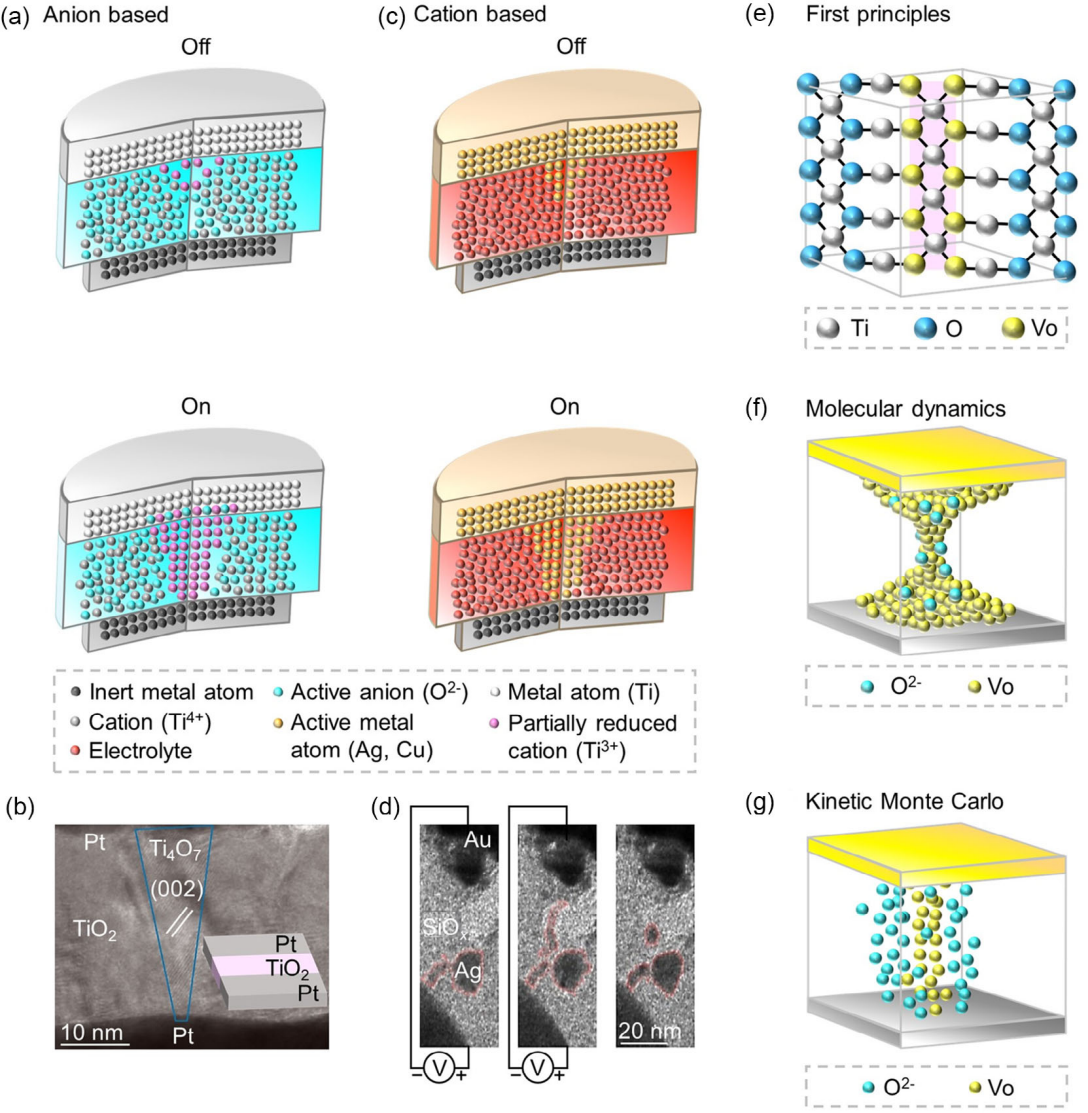
The RSM layer and physical‐modeling methodologies. a) Schematic representation of an oxygen‐vacancy filament in the Off state (top) and the On state (bottom) of an anion‐based RSM system. b) A high‐resolution transmission electron microscopy (HRTEM) image of a nanocrystalline Ti_4_O_7_ filament with a conical configuration in a Pt/TiO_2_/Pt anion‐based RSM system. Adapted with permission.^[^ ^510^ ^]^ Copyright 2010, Springer Nature. c) Schematic diagram of a metal atom filament in the Off state (top) and the On state (bottom) of a cation‐based RSM system. d) The experiment reveals an Ag filament in a planar Au/SiO_*x*_:Ag/Au cation‐based RSM system using in situ high‐resolution TEM, exhibiting the creation of a conducting Ag bridge and spontaneous withdrawal following bias removal. Adapted with permission.^[^ ^406^ ^]^ Copyright 2016, Springer Nature. e–g) Illustrations of the first‐principles approach to compute oxygen‐vacancy characters (e), the MD strategy to simulate ionic‐migration dynamics (f), and the kinetic Monte Carlo (KMC) methodology to model oxygen vacancy and interstitial‐ion distributions (g).

A variation in the electronic structure and the charge transport results when electrochemical‐redox reactions alter the chemical oxidation state of atoms in RSM layers (**Figure** **4**). Many noncrystallized dielectric materials deposited between two metal electrodes have disclosed electrically induced redox‐based switching.^[^ ^148^ ^]^ The resistive switching is a result of the redox reaction and ion migration, which are induced by electric potentials, chemical potentials, and temperature gradients over reaction coordinates, in the general theory of the resistive switching process.^[^ ^149^, ^150^, ^151^, ^152^, ^153^ ^]^ When ions are electrically or thermally stimulated, anions including oxygen ions or the corresponding oxygen vacancies become more movable compared to cations for various dielectric materials, such as perovskites and transition‐metal oxides. The variation in the metal‐cation valence in the dielectric material alters electrical conductivities when oxygen anions move toward the anode upon the application of an electric field (Figure 4a).^[^ ^34^ ^]^ An applied reverse electric field or specified temperature results in the electrochemical or electrothermal dissolution of the conduction channel, which resets the memory element to the low conductance‐off state.

Harnessing a conductive atomic force microscopy (CAFM), the configuration of a confined conduction channel of an anion‐based RSM layer was examined, which disclosed a conductivity increase for a nanosized locality after an electric field was administered to an undoped SrTiO_*x*_ system.^[^ ^154^ ^]^ Moreover, through the TEM of a Pt/TiO_*x*_/Pt system, a conical pillar‐type nanocrystallized filament of the TiO_*x*_ system, viz., the Magnéli phase, was revealed (Figure 4b).^[^ ^155^ ^]^ A distortion in the anode‐dielectric junction, i.e., the delaminated and irregular anode or top electrode with voids in dielectric layers, results from an increase in the oxygen gas at the anode that generates oxygen vacancies in the dielectric layer, as disclosed in the Pt/TiO_*x*_/Pt system.^[^ ^156^ ^]^ Through the electron energy loss spectroscopy (EELS) in TaO_*x*_, SrTiO_*x*_, and HfO_*x*_, as well as X‐ray absorption spectroscopy (XAS) in HfO_*x*_, SrFeO_*x*_, TaO_*x*_, SrTiO_*x*_, and TiO_*x*_, the composition of nanofilaments was also examined.^[^ ^157^, ^158^, ^159^, ^160^, ^161^ ^]^

The anion‐based RSM layer functions are based on conductive filament formation. The top electrode and the bottom electrode comprise inert electrodes or oxides, e.g., TiN, TaN, Pt, and Au, whereas the dielectric layer consists of metal oxides, viz., HfO_2_, ZnO, and Ta_2_O_5_. The structural ordering, migration, and accumulation of oxygen vacancies in dielectric layers result in the creation of metal oxides with unique phases and various compositions.^[^ ^162^ ^]^ When a stimulus voltage is administered to the Ta_2_O_5_ RSM layer in an initial low conductance state, oxygen vacancies are created, generating a contrast difference in the dielectric layer.^[^ ^163^, ^164^, ^165^ ^]^ The Ta_2_O_5_ layer functions as an oxygen provisioner as well as a reservoir,^[^ ^166^, ^167^ ^]^ with a specified region around the top electrode and the bottom electrode increasing with an increase in voltage.^[^ ^163^, ^168^ ^]^ The specified regions connect to generate the conductive filament. The ZnO dielectric layer, on the other hand, exhibits a more conspicuous conductive filament growth process. The displacement of oxygen vacancies in oxide‐based switching systems modulates the conductivity of the dielectric layer. The switching process is reversible and can induce oxygen atoms and oxygen vacancies to combine at the top electrode–oxide interface, resulting in conductive filament destruction. The metal electrode in RSM systems does not engage in ion migration, leading to a high programming endurance.^[^ ^169^, ^170^ ^]^

#### 2.3.2 Cation‐Based Transition

The oxidation of an electrochemically active metal including copper or silver, and subsequently, the drift of mobile cations in a solid electrolyte and the nucleation of the cation to create a conducting filament upon the reduction are involved in the redox and migration of cations that generates a conduction channel in RSM layers (Figure 4c). Different filament growth processes result from variations in redox rates and ion mobilities. The large ion mobility and the high redox rate in strong ion‐conductor RSM layers, including ternary chalcogenides, iodides, tellurides, sulfides, and selenides, lead to a filament growth toward active‐metal electrodes, while the filament growth away from the active metal electrode results for the case of the small ion mobility and the low redox rate in weak ion‐conductor RSM layers, e.g., nitrides and oxides.^[^ ^34^ ^]^ Additionally, the Ag dendrites are disclosed in aqueous electrolytes, while the planar Ag/As_2_S_3_:Ag/Ag RSM layers exhibit cation‐type filaments.^[^ ^171^ ^]^ Through an in situ TEM, the dynamic switching process of a planar Au/SiO_*x*_:Ag/Au diffusive RSM layer was revealed (Figure 4d). The Au/SiO_*x*_:Ag/Au diffusive RSM layers are different from a cation‐based RSM layer as a result of the spontaneous filament rupture following the removal of an electrical stimulus. Both the reduction and oxidation result for the Ag nanoparticle and an Ag bridge appear between metal electrodes due to the bipolar electrode phenomenon.^[^ ^172^ ^]^ The RSM layer with inert metals, along with copper or silver cation‐based RSM layers, reveals this phenomenon.^[^ ^173^ ^]^ The extended cluster of nanoparticles withdraws rapidly by the diffusion process through minimizing the interfacial energy between the dielectric and the metal upon the removal of electrical stimuli.^[^ ^174^ ^]^ Energy‐dispersive X‐ray spectroscopy (EDS) elucidates the composition of the copper or silver filament in many solid electrolytes.^[^ ^175^ ^]^ The valence‐change RSM layers, e.g., TaO_*x*_, in specified settings, disclose the cation transport process.^[^ ^176^ ^]^ Moreover, homogenous‐interfacial switching, wherein the conductance increases with an increasing device‐contact area, was demonstrated in perovskites, viz., Pr_*x*_Ca_1−*x*_MnO_*y*_, in addition to the filamentary switching. Depending on various combinations of the electrode and the RSM layer, the interfacial switching mechanism was electrochemical reaction‐based or charge trapping‐based.^[^ ^177^ ^]^

The redox reactions of a metal electrode determine the resistive switching process. The top electrode comprises active layers, e.g., Cu or Au, while inert layers such as Pt are utilized in the case of the bottom electrode. An oxidation process results when a positive stimulus is administered to the top electrode, inducing Ag atoms to be oxidized to Ag^+^ ions and electrons. Under the influence of an electric field, the Ag^+^ ions move to the bottom electrode in the RSM layer. A reduction process results, leading to the formation of an Ag‐based conductive filament, which expands and extends in the RSM layer. When the top electrode and the bottom electrode are linked, the conductance increases, which results in a change from the low conductance state to the high‐conductance state. When a negative stimulus is injected, Ag atoms in the conductive filament convert into Ag^+^ ions and electrons, resulting in the destruction of the conductive filament and an abrupt decrease in current, which leads to an alteration from the high‐conductance state to the low conductance state. The processes of the Ag‐based conductive filament growth are determined by the positions of the Ag^+^ ions and bottom‐electrode electrons during the reduction process.^[^ ^178^, ^179^ ^]^ If Ag^+^ ions are more mobile than electrons, the Ag^+^ ions will assemble at the bottom electrode, inducing the Ag‐based conductive filament to grow from the bottom electrode to the top electrode.^[^ ^173^, ^180^ ^]^ This is a typical behavior in solid electrolytes containing top electrode ions, e.g., the As_*x*_S_*y*_ lattice.^[^ ^181^ ^]^ If Ag^+^ ions exhibit a decreased mobility, it will accumulate near the top electrode, driving the Ag‐based conductive filament to grow from the top electrode to the bottom electrode. This phenomenon occurs frequently in metal oxide layers, viz., ZnO, SiO_2_, ZrO_2_, and HfO_2_.^[^ ^167^, ^182^, ^183^, ^184^, ^185^ ^]^ Moreover, recent studies have demonstrated the growth of the conductive filament in the ZnO layer from the top electrode to the bottom electrode.^[^ ^186^, ^187^ ^]^

### 2.4 Atomic‐Scale Simulations of RSM Layers

Recent studies have disclosed various physical modeling approaches for RSM layers. To attain the transition energy between different stable states and the conduction character of the stable state, ab initio calculations have been utilized.^[^ ^188^ ^]^ The ab initio calculations were harnessed to build a model with several ten to a few hundred atoms (Figure 4e). However, it is not required to include model parameters through this approach. The conventional workload for the ab initio methodology is to compute activation barriers of the ion migration. For assessing if a targeted doping framework enhances the reliability or uniformity of an RSM layer, this scheme is beneficial.^[^ ^189^ ^]^ Moreover, ab initio computations have been utilized to examine simple processes, including resistance volatility, charging and discharging events, and oxygen migration events.^[^ ^190^ ^]^ However, accurate modeling of a complicated‐stack, polycrystalline, or amorphous configuration in real RSM elements remains challenging for current ab initio strategies. As a result of the complicated calculation protocol, it is also very difficult to model the multievent and long‐term dynamic modes.

The RSM conduction mechanism studies have been performed through experiments, although debate over the physical character continues to a key barrier to large‐scale implementations. Owing to the stochasticity of conductive filament formation sites, the utilization of traditional experimental approaches to understand the conduction mechanism is difficult, necessitating theoretical computations on the atomic scale. Ab initio computations are widely utilized in RSM theoretical studies, and the computations investigate aspects including: 1) the effects of the surface and grain boundary on the electronic structure; 2) the contact barrier between the conductive filament, electrode, and dielectric; 3) the formation of charged or neutral defects in the dielectric; 4) the conductivity and stabilization energy of complicated defect configurations; 5) the energy barrier of ion electromigration; and 6) unknown phases during the conductive filament formation process, e.g., structure, chemical composition, and conductivity. The aspects (1) and (2) require the generation of an appropriate interface configuration, whereas the creation of large supercells are necessary in the case of aspects (3)–(5). Additionally, the aspect (6) utilizes ab initio computations in combination with a specified algorithm. These aspects enable the prediction of targeted conductive filament structure, elucidation of the RSM transition process, understanding of doping effects on RSM material signatures, selection of RSM layers, and guidance of experimental analysis. Enhanced computation strategies are required to alleviate the bandgap inaccuracy generated by computation methodologies and the demanding calculations induced by ultralarge primitive cells.

#### 2.4.1 Structural Signature

According to experiments, conductive filaments in RSM layers with active electrodes such as Ag and Cu comprise active metal clusters.^[^ ^191^, ^192^ ^]^ The conductive mechanism of oxide RSM layers, on the other hand, is driven by valence change. It is challenging to identify conductive filaments generated by oxygen vacancies, necessitating theoretical studies on the unique structure of the conductive filament. For instance, TiO_2_ can exhibit conductance alteration owing to the directed configuration of oxygen vacancies, according to ab initio computations, although conventional HfO_2_ and Ta_2_O_5_ simulations do not support this hypothesis. These studies illustrate how ab initio computations may be applied to a variety of RSM layers.

#### 2.4.2 Origin of Conductive Filament Formation

Recent studies have disclosed the formation or fracture process of conductive filaments in TiO_2_ RSM layers using ab initio computations and compared the model with conductive filaments in the *z* direction.^[^ ^193^, ^194^ ^]^ In the conductive filament model, a nonlocal electron distribution is generated along the conductive filament's *z* direction. The “walking” oxygen vacancies disrupt the overlapping electronic states in the fracture, viz., 2‐*V*_o_ out, configuration, rendering the electron distribution no longer a nonlocal state. A continuous chain of oxygen vacancies was also observed to induce conductivity alteration in a specified direction in TiO_2_, functioning as a conductive filament in a TiO_2_ RSM layer or driving the creation of Magneli‐phase conductive filaments. Recent works have shown the oxygen‐vacancy binding energy in monoclinic HfO_2_, which is the difference between the formation energy of concurrently incorporating various oxygen vacancies and the coexistence energy of a single oxygen vacancy.^[^ ^195^, ^196^ ^]^ Another work demonstrated the development and analysis of a coincidence site lattice grain boundary configuration for cubic HfO_2_.^[^ ^197^, ^198^ ^]^ Additionally, the results of traditional computations reveal that oxygen vacancies at the HfO_2_ grain boundary are insufficient to generate a metallic conductive channel. With the addition of Hf and Ti atoms, the Fermi energy level shifts into the conduction band, rendering the grain boundary metallic. The formation energy of interstitial Ti atoms stayed negative when Ti was utilized as an electrode, indicating that conductive filaments were generated near the grain boundary.

#### 2.4.3 Influence of Defects

The Ta_2_O_5_ is a leading oxide RSM layer with excellent switching endurance. However, due to its complex structure, Ta_2_O_5_ has not been well studied using ab initio computations. Recent works have shown the utilization of ab initio simulations to investigate the influence of interstitial Cu atoms and oxygen vacancies on the conductance of Cu/Ta_2_O_5_/Pt RSM layers.^[^ ^199^, ^200^ ^]^ In the simplest 14 atom Ta_2_O_5_ unit cells, an interstitial Cu atom was inserted, and the most stable structure was revealed when interstitial Cu atoms were set at a specified “6k” location of an oxygen plane. In the partial charge density analysis, through the Cu gap, a conductive channel arises between two neighboring Ta–O planes. A Ta_2_O_5_ supercell with five crystallized units represents four models, each of which may be computed independently. Theoretical studies have disclosed the use of HfO_2_ as thin layers in an RSM element, and that specified‐energy metastable suboxides result owing to a complicated electrode contact.^[^ ^201^, ^202^ ^]^ Pnnm HfO_3_, Pnnm Hf_2_O, and Imm2 Hf_5_O_2_ were predicted using designated algorithms, and it was demonstrated that tetragonal Hf_2_O_3_ and hexagonal HfO occurred stably at stipulated pressures.^[^ ^203^, ^204^ ^]^ Recent works have exhibited the creation of a composite structural model of tetragonal Hf_2_O_3_ embedded in HfO_2_, exhibiting that the lateral dimensions of conductive filaments may be as small as 1 nm × 1 nm and that tetragonal Hf_2_O_3_ could retain metallic conductivity.^[^ ^204^, ^205^ ^]^

#### 2.4.4 Material Selection and Experimental Guidance

The dielectric materials used in RSM layers are vital for next‐generation in‐memory computing. Since impurities influence the dynamic and thermal stability of defects, ion doping technology is frequently utilized to optimize the RSM material character. Recent works have demonstrated the utilization of Al doping into a HfO_2_ model and observed that Al impurities furnish conductive filaments a preferred growth location.^[^ ^206^, ^207^ ^]^ Theoretical studies have shown that Al‐doped HfO_*x*_‐based RSM layers are non‐stoichiometric after sputtering, oxidation, and ion implantation.^[^ ^208^, ^209^ ^]^ However, atomic layer deposition results in enhanced chemistry. Utilizing ab initio calculations, recent studies have investigated the charge state, formation energy, and migration barrier of oxygen vacancies in four high‐dielectric‐constant oxides, i.e., TiO_2_, Al_2_O_3_, Ta_2_O_3_, and HfO_2_.^[^ ^210^ ^]^ The chemical potential of oxygen exhibits a substantial influence on the RSM's retention signatures, and that the incorporation of a suitable metal layer can modulate the chemical potential of oxygen. As a result of the stable amorphous configuration, Ta_2_O_5_‐based RSM layers demonstrate enhanced programming endurance. Moreover, by analyzing physical signatures, ab initio computations can steer experiments. Recent works have calculated trap energy levels, e.g., *E*_t_ = 0.08 eV, based on TiN/HfO_*x*_/Pt experimental data. The trap‐energy level value was then utilized in a conduction mechanism analysis.^[^ ^211^ ^]^ Another work disclosed that a recurrent conductance‐alteration phenomenon necessitates choosing an electrode that allows the active layer to exhibit a Fermi level between those of the two electrodes.^[^ ^212^, ^213^ ^]^ When Pt and Mo are utilized as metal electrodes, ab initio calculations further reveal that the Fermi levels of Pt and Mo are in the upper and lower defect bands, indicating that Pt Mott transitions occur independently of Mo.

#### 2.4.5 Ion Migration and Variabilities

To investigate the ion migration process in RSM layers and the associated variabilities, dynamic simulation methodologies, i.e., KMC and MD simulations, are effective tools (Figure 4f,g).^[^ ^37^ ^]^ The reactive MD approach with a charge equilibrium protocol was utilized to examine the generation of the stable filament and metastable atom chains in a cation‐based RSM layer.^[^ ^214^ ^]^ The event‐driven and stochastic‐type methodology, viz., the KMC mode, computes only in the time between two events and is not required to perform computations over each period. As a result, the KMC approach has been utilized for understanding processes with different timescales, e.g., the resistive switching process over few nanoseconds and the long‐term data retention over several years.^[^ ^215^ ^]^ For the traditional KMC strategy, the disadvantage is that the methodology involves various assumptions and model parameters. Moreover, the MD approach adheres to Newton's principles of motion and has attracted interest since the emergence of the digital computer. The MD methodology is required for stable systems, including large systems. MD simulations facilitate achieving equilibrium, furnishing a distinct route to solutions. MD approaches, as compared with discrete lattice‐based methodologies, utilize consistent conditions and minimize design issues. Although MD simulations are computationally demanding, the MD approach exhibits no intrinsic constraints. Due to interface concerns, intrinsic variations in small systems, and inhomogeneities, e.g., density or temperature gradients, traditional MD simulations encounter difficulties. Slow relaxation processes result in increased timescales by orders of magnitude and are induced in polymer‐melt diffusion blocks, glassy states, and spontaneous spatial‐ordering entities.

### 2.5 MTM‐Layer Switching Ability

**Figure 5:**
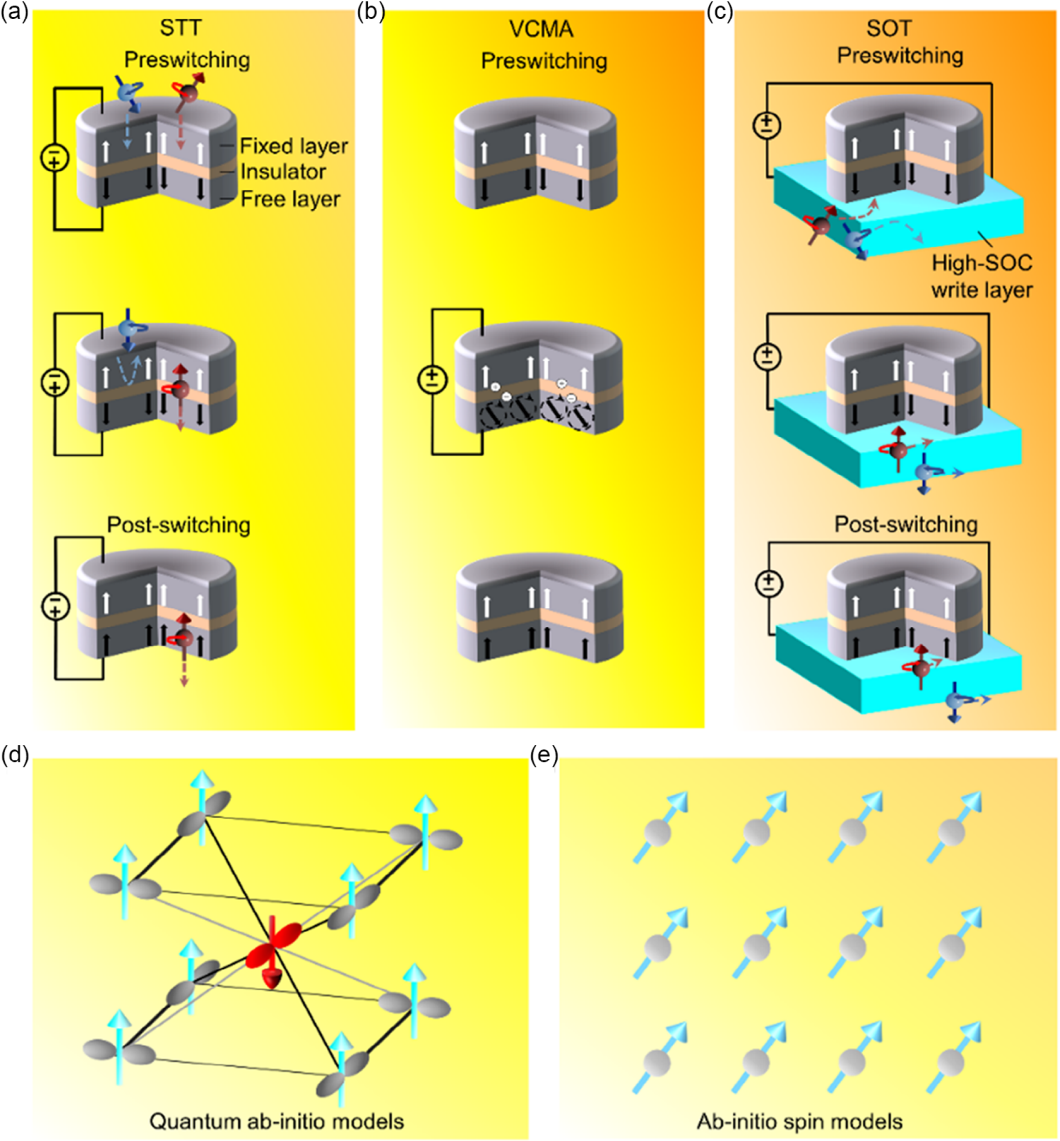
MTM layers and modeling strategies. a) The spin‐transfer torque (STT) process. Electrons are spin polarized by the magnetic moment in the fixed layer when the electron pass through the magnetically fixed layer. Moreover, electrons oriented in the opposite direction to that of the fixed layer are reflected, while the electron oriented in the same direction as that of the magnetically fixed layer flows through the tunneling barrier. The spin‐angular momentum of electrons that flow through the tunneling barrier are transferred to the magnetic‐free layer moments, thus altering the free‐layer magnetization or material state. b) The voltage‐controlled magnetic anisotropy (VCMA) mechanism. The charge depletion or accumulation at the magnesium oxide‐magnetic free layer interface alters the anisotropy of the magnetic‐free layer when an input voltage is administered across the magnetic‐tunneling junction, resulting in a precession, viz., oscillation, of the free‐layer magnetization. The free‐layer magnetization or material state is switched when the length of the input signal is half that of the precession process. c) The SOT mechanism. The magnetic‐tunneling junction is deposited on top of a high spin–orbit coupling (SOC) layer, to enable this process. Electrons with a specified spin direction aggregate near the top of the SOC layer, whereas the electron with an opposite spin direction accrues close to the bottom of SOC layers, when a current flows through the same SOC layer. The electrons assembled adjacent to the top of the SOC layer pass into the magnetic‐free layer and transfer the spin momenta to the same magnetic‐free layer, thus altering the free‐layer magnetization or material state. d,e) Simulation tools, viz., (d) the quantum ab initio model and (e) ab initio spin models, are utilized for MTM development, e.g., phenomena, materials, and other areas, to understand the material signature, and consequently steer the material optimization.

The magnets have been the foundation of storage devices since the development of hard disk drives, and magnetic‐tunneling memory (MTM) layers have been utilized for next‐generation edge computing.^[^ ^216^ ^]^ Combining the MTM layer in a magnetic‐tunnelling‐junction (MTJ) configuration with the CMOS logic system is a recent approach.^[^ ^217^ ^]^ The MTJ configuration, based on two ferromagnetic layers that sandwich a thin insulator as a tunneling barrier, discloses a reference or fixed magnetic layer with a specified magnetization direction, and another magnetic‐free layer that can be switched between two different directions (**Figure** **5**). For the CMOS integration, various works have been performed for enhancing MTJ configurations. The MTJ configuration with a giant magnetoresistive spin valve containing the Cu between ferromagnetic layers has been improved to MTJ configurations with an Al_2_O_3_ tunneling barrier.^[^ ^218^ ^]^ By altering the tunneling barrier from the amorphous Al_2_O_3_ to a crystalline MgO, an enhancement in the tunneling magnetoresistance ratio was also achieved.^[^ ^219^ ^]^ Furthermore, the downscalability was facilitated by modifying the magnetic anisotropy of the ferromagnetic layer from the in‐plane direction to the out‐of‐plane direction.^[^ ^219^ ^]^ From the magnetic field switching effect created by an electrical current to harnessing quantum effects, including spin‐transfer torque (STT), voltage‐controlled magnetic anisotropy (VCMA), and spin–orbit torque (SOT), the switching process of the ferromagnetic layer has advanced through various development phases.

#### 2.5.1 Spin Transfer Torque‐Based Transition

To modulate the magnetic state of the free layer, the MTM layer based on the STT effect utilizes the spin‐polarization phenomenon.^[^ ^220^ ^]^ Most electrons become spin polarized by the magnetization direction of the fixed layer if the electrons pass through the magnetically fixed layer.^[^ ^221^ ^]^ The electrons then render the spin‐angular momentum to the free magnetic layer, thus altering the magnetization direction of the free layer to that of the fixed layer, viz., the parallel mode results (Figure 5a). The remaining electrons with a spin polarization opposite to that of free layers move toward the free layer when the current direction is altered.^[^ ^218^ ^]^ As a result, the free layer switches and becomes antiparallel to the fixed layer. Currently, MTM layers with the STT effect are utilized by major foundry corporations for achieving memory applications since it is a technically mature technology.^[^ ^222^ ^]^ Moreover, at ambient temperature, the magnetoresistance ratios of AlO‐based MTJ configurations are below 100% owing to an amorphous tunnel barrier that enables distinct Bloch states with various spin polarizations to tunnel through. For conventional transition‐metal ferromagnets, this leads to a tunneling spin polarization of less than 0.5. When the tunnel barrier is crystallized, e.g., MgO (001), electrons coherently tunnel through barriers, with only the $\Delta_{1}$ states predominately tunneling over the barrier.^[^ ^223^, ^224^ ^]^ This is due to the fully spin‐polarized $\Delta_{1}$ states of body‐centered‐cubic Fe (001), which results in potentially large magnetoresistance ratios, i.e., above 1000%.^[^ ^225^, ^226^ ^]^ Recent studies demonstrated the utilization of molecular beam epitaxy to construct high‐quality fully epitaxial Fe (001)/MgO (001)/Fe (001) MTJ configurations with magnetoresistance ratios of as high as 180% at room temperature.^[^ ^227^, ^228^ ^]^ Furthermore, experiments have exhibited the use of sputter deposition to create MTJ configurations with a strongly orientated polycrystallized MgO (001) barrier, resulting in high magnetoresistance ratios of up to 220% at ambient temperature.^[^ ^229^, ^230^ ^]^

Despite the high magnetoresistance ratios, conventional MgO‐based MTJ configurations meet difficulties in application. The MTJ configuration should be grown on an antiferromagnetic‐synthetic antiferromagnet multilayer with a face‐centered cubic (111) structure and threefold in‐plane crystallographic symmetry. Traditional MgO (001)‐based MTJ configurations with fourfold in‐plane symmetry, on the other hand, cannot be grown on face‐centered‐cubic (111) structure. Recent works have disclosed the creation of CoFeB/MgO/CoFeB MTJ configurations to alleviate crystal growth concerns. At room temperature, the work revealed a magnetoresistance ratio of more than 200%.^[^ ^231^, ^232^ ^]^ When the B concentration is large, amorphous CoFeB layers result. By sputtering under optimized conditions, a highly textured MgO (001) layer grows on amorphous CoFeB. Solid‐phase epitaxy occurs when CoFeB layers crystallize from MgO (001) interfaces when annealed above 520 K.^[^ ^233^, ^234^ ^]^ The annealing approach generates fourfold symmetry textured CoFeB (001)/MgO (001)/CoFeB (001) layers that may be grown on a variety of bottom structures or substrates. Owing to the fully spin‐polarized $\Delta_{1}$ bands at *E*_f_, the layers exhibit large magnetoresistance ratios up to several hundred percent at ambient temperature, similar to body‐centered‐cubic Fe (001) structure. These MTJ configurations are widely utilized in current device platforms such as STT‐MTM and magnetic sensors.

#### 2.5.2 Voltage‐Controlled Magnetic Anisotropy‐Based Switching

To induce switching, the MTM layer with the VCMA effect utilizes the capacitive signature of MgO barrier layers. The charge accumulation or depletion at the MgO‐magnetic free layer interface alters the electronic occupation of atomic orbitals if an electrical voltage is administered across the VCMA‐based MTJ configuration.^[^ ^235^ ^]^ This process results in a modification in the total magnetic anisotropy of the free layer (Figure 5b). The free layer discloses a magnetization orientation in the in‐plane direction when a large voltage is injected to the MTJ configuration, as depicted by the Landau–Lifshitz–Gilbert equation.^[^ ^236^ ^]^ To alter the magnetization orientation of the free layer along the in‐plane direction, a subsequent voltage pulse with a duration half of that required to exhibit the magnetization orientation in the in‐plane direction is utilized. To inhibit the STT effect and minimize the current, recent studies have demonstrated a thick MgO barrier layer with a large resistance–area product.^[^ ^237^ ^]^ The Cr‐buffer and MgO‐seed layers have also been enhanced for the epitaxial Fe/MgO/Fe growth to boost the VCMA phenomenon.^[^ ^238^ ^]^ Moreover, the feedback of electronic orbitals to an administered voltage stimulus strengthens when the Mg layer is incorporated at CoFeB–MgO interfaces.^[^ ^239^ ^]^ The thermal barrier layers, including W and Mo, have been further examined to inhibit the capping or seed layer from diffusing into CoFeB/MgO layers, to enable the compatibility of voltage‐controlled MTJ configurations with the thermal budget of industrial‐CMOS back‐end‐of‐line (BEOL) processes.^[^ ^240^ ^]^

In the VCMA effect, perpendicular magnetic anisotropy is vital for enhancing the thermal stability of room‐temperature nanomagnets. At the interfaces of 3d transition metal ferromagnets and heavy nonmagnetic metals, strong perpendicular magnetic anisotropy is observed.^[^ ^241^, ^242^ ^]^ Traditional Co–Pd, Co–Pt, and Co–Au heterostructures, on the other hand, exhibit substantial Gilbert damping, rendering the heterostructure unsuitable for current‐induced magnetization switching and other magnonics applications. Since there is no oxide or dielectric layer in the conventional heterostructures, electric field cannot influence interfacial perpendicular magnetic anisotropy. The perpendicular magnetic anisotropy may also be observed at the interfaces of 3d‐ferromagnets, i.e., Fe, Co, and the alloys, and MO_*x*_, viz., M = Ta, Al, Mg, and Ru. The decreased damping constant and high tunneling magnetoresistance ratio of MgO‐ and CeFeB‐based heterostructures have attracted substantial interest.^[^ ^243^, ^244^, ^245^ ^]^ Fe's out‐of‐plane $3d_{z^{2}}$‐orbitals connect with O's 2p_*z*_‐orbitals at the CoFeB–MgO interface, resulting in charge transfer. This lowers the population of electrons in out‐of‐plane orbitals, which leads to a perpendicular magnetic anisotropy through ferromagnet SOC. An electric field applied at the metal–oxide interface influences the number of electrons in Fe's 3d‐orbitals in connection with in‐plane orbitals, altering bonding strength and generating a substantial variation in interfacial perpendicular magnetic anisotropy.^[^ ^246^, ^247^ ^]^ Owing to the constrained penetration depth of the electric field, this phenomenon is limited to ultrathin ferromagnetic films. According to alternative concepts, the inhomogeneous electric field at the metal–oxide interface connects to the quadrupole of the electronic orbital in ferromagnets, which links to the magnetic dipoles and determines interfacial perpendicular magnetic anisotropy.^[^ ^248^, ^249^ ^]^ Rashba SOC may generate interfacial perpendicular magnetic anisotropy at the ferromagnet–oxide interface.^[^ ^250^, ^251^ ^]^ The Rashba SOC is altered by the electric field, which modulates interfacial perpendicular magnetic anisotropy. Experiments showed that MgO's piezoelectric signature can modify interfacial perpendicular magnetic anisotropy via magnetoelastic coupling.^[^ ^249^, ^252^ ^]^ These processes influence interfacial perpendicular magnetic‐anisotropy modulation at the same time, allowing for high‐speed functioning of VCMA magnoics devices with high cycle endurance with negligible atomic movement or chemical interactions.

#### 2.5.3 SOT‐Based Transitions

The surface spin accumulation from metal layers with a large SOC is utilized for the MTM layer with the SOT effect, which modulates efficiently the magnetization adjoining to SOC metal layers.^[^ ^253^ ^]^ The electrical current passes through a distinct high‐SOC metal layer that is below the MTJ configuration, in contrast to that of traditional STT‐based MTJ configurations. The range of material systems utilized for enhancing the programming‐power efficiency broadens and an increase in the MTJ cycling endurance results, since the current does not flow through the MTJ configuration (Figure 5c). The Rashba interfaces and bulk‐heavy metals are key high‐SOC material systems demonstrated in recent reports.^[^ ^254^ ^]^ Moreover, through SOC mechanisms such as the Rashba effect, in‐plane current induces effective magnetic fields and generates torques on magnetization.^[^ ^255^, ^256^ ^]^ Inversion symmetry is broken at the heavy metal–ferromagnet interface, resulting in an asymmetric crystal field potential and a net electric field *E*_*i*_ perpendicular to the interface. The *E*_*i*_ points from heavy metals to ferromagnets for higher work functions of the heavy metal than that of the ferromagnet. The electric field becomes an effective magnetic field, thus coupling to the magnetic moment of the electrons across the interface. In magnetic heterostructures with broken inversion symmetry, this is known as the Rashba effect. Additionally, the Rashba effect has been demonstrated in a variety of materials, such as metal surfaces, the interface between heavy metal and ferromagnet, and semiconductor heterostructures.^[^ ^257^, ^258^, ^259^, ^260^ ^]^ The spin Hall effect, which is created by bulk SOC, transforms charge currents into orthogonally flowing spin currents that transfer angular momentum and apply torque on magnetization.^[^ ^261^, ^262^ ^]^ With the in‐plane external magnetic field perpendicular to the current direction, SOT has been reported in systems with Cu at the heavy metal–ferromagnet interface and symmetric heavy‐metal–ferromagnet–heavy‐metal magnetic heterostructures,^[^ ^263^, ^264^, ^265^ ^]^ with low magnetic response.^[^ ^266^, ^267^ ^]^

### 2.6 Atomistic Modeling of MTM Layers

The intrinsic magnetic character of MTM layers vital to spintronic applications have been predicted well through ab initio quantum‐mechanical models utilizing the DFT (Figure 5d).^[^ ^268^ ^]^ The DFT calculations were also combined with the nonequilibrium Green's function methodology to understand the conduction signature of a complex system. However, traditional computations lack various key aspects that affect spintronic functionalities substantially, e.g., realistic magnetization dynamics, finite temperatures, and defects, and are theoretical. Atomistic spin dynamics (ASD) models are required for alleviating the disorder and finite‐size effects for modeling the MTM layer.^[^ ^269^ ^]^ The model was utilized to examine the dynamic magnetic signature in various systems, and these models elucidated the origin of the local disorder and depicted thermal‐spin waves well (Figure 5e). The models are appropriate for calculating the dynamic response of magnetic materials, viz., the switching duration in MTM layers with the STT effect, and the spin accumulation and spin currents at the interface on the nanometre scale, when combined with spin‐transport models.^[^ ^270^ ^]^

#### 2.6.1 Ultrafast Magnetization Dynamics

The discovery of nickel's sub‐picosecond magnetic response to femtosecond stimuli led to the studies of ultrafast magnetization dynamics.^[^ ^271^, ^272^ ^]^ This discovery has rendered it accessible to demagnetize, generate spin currents, and switch magnetic polarity rapidly.^[^ ^273^, ^274^, ^275^, ^276^, ^277^ ^]^ Magnetization dynamics have also been explored by alternate rapid excitations such as picosecond electric and spin currents.^[^ ^278^, ^279^ ^]^ Owing to its distinctive deterministic heat‐induced magnetization switching, ferrimagnetic materials, specifically amorphous GeFeCo alloys, are vital in the field.^[^ ^280^, ^281^ ^]^ Mn_2_Ru_*x*_Ga has recently shown thermal switching as well.^[^ ^282^, ^283^ ^]^ Understanding the switching in traditional GdFeCo ferrimagnetic alloys, however, is still difficult.^[^ ^284^, ^285^ ^]^ The ASD simulations are typically utilized to discern experimental results.^[^ ^286^, ^287^ ^]^

The electron system can be excited by an input stimulus that extends a few femtoseconds on the timescales of the exchange interaction, enabling studies of the underlying physics controlling switching. Electrons are accessible to the electric field when a metallic ferrimagnetic thin film is exposed to an input stimulus, which heats up the electron system. For up to a few picoseconds, the temperatures of the phonon and electron are isolated. The so‐termed two‐temperature model (2TM), which can be expressed as two coupled differential equations, captures this phenomenon.^[^ ^288^, ^289^ ^]^ The ability of a femtosecond input stimulus to alter the magnetization polarity in specified systems has been demonstrated through ASD models and experiments.^[^ ^280^, ^290^ ^]^ Although the FeCo and Gd spin sublattices are antiferromagnetically coupled, the sublattices exhibit parallel alignment, or a “transient ferromagnetic‐based state”, during the switching operation. These results are demonstrated by recent studies using element‐specific time‐resolved femtosecond X‐ray magnetic circular dichroism.^[^ ^291^, ^292^ ^]^ Picosecond switching times have been achieved in recent works using magnetic tools at the micro‐ and nanometer lengthscales.^[^ ^284^, ^293^ ^]^ Due to its ability to switch using heat generated by picosecond electric currents and optical pulses, this variant of magnetic switching exhibits great potential for future applications.^[^ ^278^, ^294^ ^]^ According to ASD simulations, the switching path induced by femtosecond to picosecond heating traverses the same route.^[^ ^295^ ^]^

#### 2.6.2 Spincaloritronics

Spincaloritronics is the investigation of the physical effects induced by the coupling of spin and heat.^[^ ^296^, ^297^ ^]^ In various materials, spincaloritronics comprises phenomena, viz., spin Peltier effect, spin Seeback effect, and spin Nernst effect.^[^ ^298^, ^299^, ^300^, ^301^, ^302^ ^]^ Magnetic insulators are utilized to explore magnon‐driven events in the absence of charge transport convolution. Most “ferromagnetic” magnetic insulators, on the other hand, are ferrimagnets. Traditional theoretical studies in this domain neglect the existence of at least two antiparallel sublattices and model materials as ferromagnets by assuming a single magnon band with a $\hbar \omega \approx k^{2}$ dispersion. ASD modeling is utilized to examine what these approximations could overlook in ferrimagnetic materials. Additionally, ASD simulations were utilized to investigate the magnonic spin Seeback effect in a ferrimagnet, which is the response of magnetization to a temperature gradient. A conventional two sublattice ferrimagnet was used in a temperature step to furnish information about the spatial distribution of sublattice magnetization.^[^ ^285^, ^303^ ^]^ In the absence of a temperature step, a stationary nonequilibrium magnon accumulation occurs, corresponding to the derivative of equilibrium magnetization $\partial m_{z}/\partial T$.

Modeling in spincaloritronics has focused on magnon polarization in ferrimagnets, which is connected to spin rotation or the angular momentum of a magnon mode. Magnons exhibit a single circular polarization in a ferromagnet, which corresponds to anticlockwise magnetic moment rotation. Both anticlockwise and clockwise polarizations appear in uniaxial antiferromagnets; however, the magnon modes degenerate, which renders conventional polarization measurement challenging. Anticlockwise and clockwise polarizations occur in ferrimagnets owing to opposing sublattices, but the exchange field distinguishes the modes, rendering clockwise magnons more energetic. Besides, magnon polarization influences the spin Seeback effect in gadolinium iron garnet (GdIG), with two sign alterations observed as temperature increases.^[^ ^304^, ^305^ ^]^ At the magnetization compensation point, where sublattice reverse in the applied field, one alteration is anticipated. At low temperatures, the sign alteration does not correspond to macroscopic changes in conventional ferrimagnet. According to theory and ASD modeling, the sign alteration is induced by varying thermal occupation of magnon modes with varied polarization. Moreover, since the initial understanding of magnons, theoretical studies have examined magnon polarization, but the magnon polarization has not been physically measured. For the first time, polarized inelastic neutron scattering was utilized, which was supported by ASD modeling.^[^ ^306^, ^307^ ^]^ Because the scattering cross section is minimal in the required experimental geometry, experiments are challenging. Prior estimates utilizing ASD modeling revealed a limited success. A second measurement was successful, and there was excellent agreement between the ASD calculations and the experimental findings. Considering magnon transport is influenced by polarization, the polarization of various magnon modes may be advantageous in spintronics applications.^[^ ^308^, ^309^ ^]^

Experiments to stimulate the yttrium iron garnet (YIG's) high‐frequency magnon modes have been utilized.^[^ ^310^, ^311^ ^]^ Recent studies have demonstrated the generation of long‐wavelength THz phonons in YIG using THz input stimulus and the measurement of magnetization alterations using the magneto‐optical Faraday effect.^[^ ^312^, ^313^ ^]^ The studies demonstrated a decrease in sublattice magnetization on picosecond timescales that extends for microseconds before reverting to normality. The movement of light oxygen ions generated by the activation of infrared active THz phonons induces variations in the super exchange between Fe atoms owing to alterations in bond angles and lengths. To assess the findings, ASD computations were used, indicating that net magnetization stays consistent notwithstanding decreasing sublattice magnetization owing to the isotropic exchange interaction that conserves spin angular momentum. In addition, on the sub‐picosecond timescale, ultrafast magnetization dynamics and spincaloritnics are integrated to examine characters including the spin Seeback effect. Recent works have exhibited the heating of the Pt of a YIG‐Pt bilayer using a femtosecond stimulus, demonstrating a large temperature variation between the magnons and phonons of the YIG and the hot electrons in the Pt.^[^ ^314^ ^]^ The goal was to investigate how rapidly the spin current across the YIG‐Pt interface occurs as a result of electron‐magnon scattering. A dynamical theory was generated which required signatures including the YIG's frequency‐dependent spin susceptibility. This was computed with ASD simulations and an extensive YIG model, revealing that the magnetic system responds quickly owing to the lack of inertia. The theory estimated interfacial sd‐exchange coupling and spin mixing conductance, which agreed well with previous estimates in the literature.

#### 2.6.3 Magnetic Textures

Owing to the potential for data storage and processing, spin textures including skyrmions and domain walls are an important research topic. Beyond considering ferrimagnets as ferromagnets, ferrimagnets have not been well studied. The key distinction between ferro and ferrimagnets is the textural dynamics induced by magnetization compensation sites and angular momentum. At the angular compensation point, ferrimagnets function as antiferromagnets and move at high speeds due to torque compensation. To anticipate and understand domain wall dynamics in the presence of external fields, analytical theory based on macrospin methodologies was utilized.^[^ ^279^, ^315^ ^]^ Ferrimagnetic domain wall's precession and velocity may be represented in terms of gyromagnetic ratio and effective damping. Besides, computational models are required for understanding thermal effects including magnetic texture migration under heat gradients. ASD modeling has been effective in this context. Recent studies investigated domain walls in ferrimagnets treated with heat gradients.^[^ ^316^, ^317^ ^]^ The works demonstrated that domain walls in ferro‐ and antiferromagnets migrate toward hot regions.^[^ ^318^, ^319^ ^]^ However, the domain wall approaches the cold region above the Walker breakdown if the temperature is smaller than the angular‐momentum compensation threshold. Contrary to previous assumptions, the studies revealed aberrations in ultrafast stimulus studies on GdFeCo, exhibiting domain walls migrating away from the heated zone.^[^ ^320^ ^]^ This indicates the importance of ferrimagnet ASD modeling, as these characters are difficult to distinguish in experiments. The work also exhibited a “torque compensation point”, where ferrimagnetic domain walls move in the same way as antiferromagnetic domain walls, with inertia free motion and no Walker breakdown. Furthermore, the magnetic skyrmion domain aims to modulate the skyrmion Hall effect, which describes the transverse motion of an askyrmion when pushed by a specified stimulus. Skyrmions exhibit no Hall effect in antimagnets owing to Magnus force cancellation.^[^ ^321^, ^322^ ^]^ Recent GeFeCo experiments revealed that the skyrmion Hall effect in ferrimagnets was approximately zero at the angular‐momentum compensation point.^[^ ^323^, ^324^ ^]^ Magneto‐optical Kerr effect (MOKE) imaging and ASD simulations furnish this evidence.

## 3 MM System Characters

**Figure 6:**
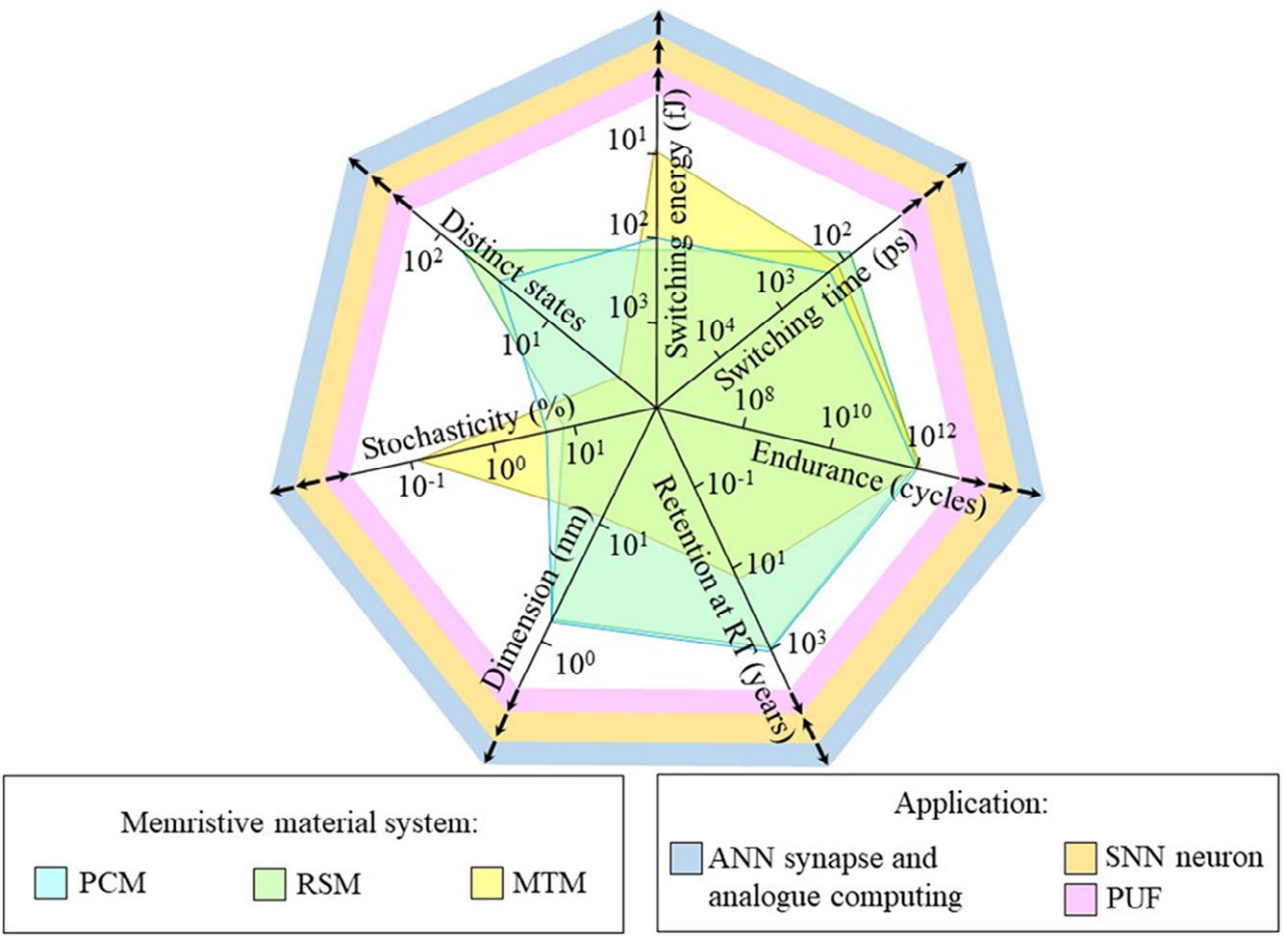
MM system signatures and application specifications. The plot compares the characters of three different types of MM systems and their influence on applications such as analogue in‐memory computing, spiking neural network (SNN) neurons, physical unclonable functions (PUFs), and artificial neural network (ANN) synapses. Short switching times, small dimensions, low programming energy, and excellent endurance are beneficial for the sample applications shown in this survey. PUFs are facilitated through a high degree of randomness, whereas SNN neurons and ANN synapses require minimized stochasticity. Furthermore, SNN neurons utilize spontaneous, rapid conductance relaxation, while ANN neurons and PUFs are assisted by prolonged retention.

The MM conductance alterations are determined by distinct physics, but the MM systems exhibit similar material signatures (see **Table** **1** and **Figure** **6**), allowing for a wide range of computing applications. The goal of universal memory is to optimize memory hierarchy, minimize data‐shuffling time delay, and decrease energy consumption. Commercial memristive elements have been utilized since 2005, but improvements, e.g., enhanced data retention, power consumption, switching time, storage capacity, and cycle endurance, are required to achieve universal memory.

**Table 1:** MM system signatures' maximum or minimum values

MM system	Min. switching energy [fJ]	Min. switching time [ps]	Max. endurance [cycles]	Max. retention at RT [years]	Min. dimension [nm]	Min. stochasticity [%]	Max. no. of distinct states
PCM	100–1000	500–700	10^7^–10^12^	>1000	2–5	5.00–10.00	16–32
	(refs. [328, 511])	(refs. [129, 512])	(refs. [353, 513])	(refs. [340, 514])	(refs. [327, 515, 516])	(refs. [329, 372])	(refs. [372, 376, 517])
RSM	115–144	85–200	10^6^–10^12^	>1000	2–18	9.65–10.00	20–64
	(refs. [325, 518])	(refs. [330, 519])	(refs. [520, 521])	(refs. [341, 522])	(refs. [360, 523])	(ref. [524])	(refs. [377, 525])
MTM	10–50	200–225	10^10^–10^12^	10–20	10–22	0.29–0.40	2–10
	(refs. [336, 526])	(refs. [338, 527])	(refs. [356, 528])	(refs. [244, 529])	(refs. [530, 531])	(ref. [356])	(refs. [243, 532, 533])

### 3.1 Switching Time and Energy

Low programming energy and quick switching time are advantageous for many computing types, including the computing mode requiring frequent element programming, such as digital computing. The reset switching process exhibits the largest effect on the switching energy in RSM and PCM layers. 100 fJ is the reset energy achieved for RSM layers,^[^ ^325^, ^326^ ^]^ but in the case of the PCM layer, the reset energy is proportionate to switching volume.^[^ ^327^, ^328^, ^329^ ^]^ In conventional RSM layers, switching times are restricted by the redox‐reaction rate and ion‐migration speed, but the switching time is limited by the nucleation and growth rates in the scenario of the PCM layer. The active‐entity choice, applied electric field, and temperature influence both types of MM systems. RSM layers based on nitrogen‐vacancy migration, for instance, exhibit the shortest switching time of 85 ps,^[^ ^330^, ^331^ ^]^ while the GeSbTe PCM layer discloses a crystallization pulse of 500 ps.^[^ ^72^, ^332^ ^]^ Scandium‐doped antimony telluride PCM layers have also been examined to speed up the crystallization process.^[^ ^129^, ^333^ ^]^ As a result of the mild atom movement, MTM layers require low programming energy and short switching time. Utilizing a designed free layer, STT MTJ configurations reveal a switching time of 10 ns and a 50 μA programming current.^[^ ^334^, ^335^ ^]^ A VCMA MTJ configuration with a diameter of 50 nm has been demonstrated to exhibit the lowest energy of 10 fJ among all MM systems.^[^ ^336^, ^337^ ^]^ Furthermore, a similar STT MTJ configuration discloses a switching time of 200 ps and, with the use of low‐voltage stimuli, close to SRAM capabilities.^[^ ^338^, ^339^ ^]^

### 3.2 Endurance and Retention

Data stability is important for the integration of in‐memory computing, artificial neural networks, and hardware elements, whereas rapid conductance decay may be utilized to imitate specified brain processes and create random numbers. The increase in the potential barrier between physical states increases switching energy while simultaneously increasing conductance state retention, in accordance with the Arrhenius temperature dependency. RSM layers with Ta–O conduction channels or high crystallization‐temperature PCM layers with Ge enrichment and nitrogen doping can attain room‐temperature retention of more than 10 years.^[^ ^340^, ^341^, ^342^ ^]^ The thermal stability factor of MTJs is proportional to the energy barrier and scales with element volume. Recent works have demonstrated STT MTJ arrays with high thermal stability factors, viz., data retention of more than ten years.^[^ ^343^, ^344^, ^345^ ^]^ High cycle switching endurance is important in computing, specifically in applications that require consistent programming, such as digital in‐memory computing. Element endurance failure can be induced by structural fatigue in traditional RSM and PCM layers. For conventional RSM layers, the structural fatigue comprises filament overgrowth, aberrant reactions, or filament atom loss.^[^ ^346^, ^347^ ^]^ Close to ≈10^12^ programming cycles were achieved in a thermodynamically stable RSM layer.^[^ ^348^ ^]^ The prototypical PCM‐layer endurance is associated with phase segregation, mechanical stress, and electromigration induced by density variations.^[^ ^349^, ^350^, ^351^, ^352^ ^]^ Increased endurance can be attained by modifying the composition, element structure, and film deposition process. Up to ≈10^12^ programming cycles were attained in a GeSbTe PCM layer.^[^ ^353^, ^354^ ^]^ Due to decreased structural fatigue and low atomic displacement during programming, MTM layers demonstrate a high switching endurance. After reactive‐ion‐etching damage alleviation, STT MTJ configurations exhibit ≈10^10^ programming cycles, as well as intermediate thermal stability.^[^ ^345^, ^355^ ^]^ Furthermore, SOT MTJ configurations with CoFeB/MgO/CeFeB stacks show programming cycles of ≈10^12^.^[^ ^356^, ^357^ ^]^

### 3.3 Stacking Ability and Compactness

High element density improves mobility and lowers computing platform costs, rendering a general benefit for applications using a high number of MM systems, such as neural networks and in‐memory computing. Owing to the localized conduction channels, RSM layers are scalable. HfO_2_‐based RSM layers with an element size of 10 nm demonstrated quick and stable performance.^[^ ^358^, ^359^ ^]^ Moreover, HfO_2_/TiO_*x*_ RSM crossbars with low current have been disclosed.^[^ ^360^, ^361^ ^]^ TaO_*x*_‐type RSM layers have also been integrated in an ≈7 Mbit array on a 22 nm transistor low‐power node.^[^ ^362^ ^]^ Carbon nanotube electrodes with a contact size of 5 nm were utilized to evaluate PCM layers.^[^ ^327^, ^363^ ^]^ The decrease in switching energy facilitated the incorporation of an 8 Gb PCM layers on a 20 nm‐node system utilizing diode‐based accessing elements.^[^ ^364^ ^]^ The stacking ability of PCM and RSM layers has been demonstrated with the fabrication of 3D cross‐point memory and an eight‐layer 3D vertical TiN/HfO_2_/TaO_*x*_/Ti/TiN/W array, respectively.^[^ ^365^, ^366^, ^367^ ^]^ For enhanced retention and state number, traditional MTM layers using domain switching require a large footprint. However, this necessitates a trade‐off between element characters and entity density. STT MTJ configurations have exhibited megabit integration on 22 or 28 nm‐technology nodes,^[^ ^368^, ^369^ ^]^ with the ability to scale down to the 10 nm‐technology node.^[^ ^370^, ^371^ ^]^

### 3.4 Randomness and State Number

Various computer workloads require different degrees of data retention capability. The number of bits represented by MM systems determines performance in neural networks and analog computing. Higher number of physical states or a larger ratio of conductance range to programming variability result in more effective bits per element. However, although digital computing utilizes binary states, cybersecurity applications require stochasticity. Owing to atom rearrangements in the switching process, traditional RSM and PCM layers may exhibit large and medium programming variabilities, respectively. However, 1D channels in epitaxial dielectrics, confinement nanolayers, and iterative‐programming approaches minimize the programming variability,^[^ ^372^, ^373^, ^374^, ^375^ ^]^ resulting in ≈64‐level and 32‐level functional conductance in the corresponding RSM and doped‐GeSbTe PCM layers.^[^ ^376^, ^377^ ^]^ Domain phenomena are utilized in MM layers, with MTJ configurations exhibiting a single domain and minimized programming variability. Due to precessional switching, conventional VCMA MTJ configurations reveal high programming error rates,^[^ ^378^, ^379^ ^]^ whereas a small programming error rate throughout a large temperature range is disclosed in the case of STT MTJ configurations.^[^ ^368^, ^380^ ^]^

## 4 MM‐Based In‐Memory Computing

Brain‐inspired computing, which is inspired by brain function, is a vital technology for artificial intelligence. The neuromorphic and analogue computing technologies process information in a brain‐inspired way by using the physical signatures of MM systems. Cybersecurity applications utilize hardware roots of trust based on process‐induced differences in electronic hardware for cryptographic data. The MM systems furnish a low‐cost and readily available alternative for random number generators (RNGs), physical unclonable functions (PUFs), and hardware identities, owing to the inherent randomness in digital operations.

### 4.1 Brain‐Inspired and Analog Computing

**Figure 7:**
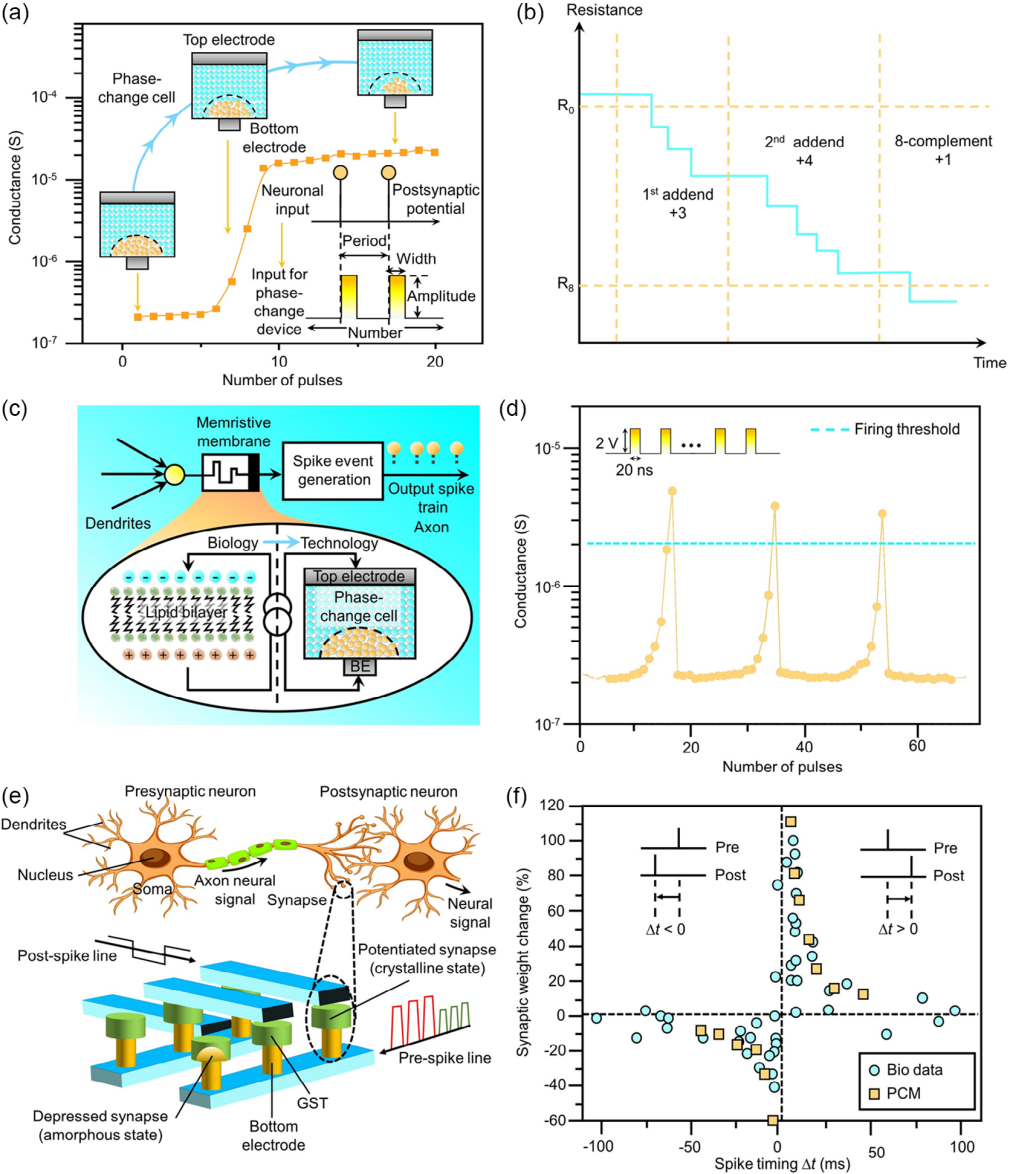
MM‐powered brain‐inspired and analog computation. a) Variation in the output conductance of an MM system for different stimulus numbers. An increased number of stimuli results in an increasing structurally ordered volume, leading to an increase in the output conductance value. b) The arithmetic addition of addends “three” and “four” through the stimulus aggregation in an MM element. c) An integrate‐and‐fire neuron, wherein the integration was performed based on accruing input spikes in an MM unit. d) The synaptic potentiation utilizing the gradual structural ordering of an MM entity. a,c,d) Adapted with permission.^[^ ^398^ ^]^ Copyright 2016, Springer Nature. e) The schematic diagram compares illustrations of biological neurons with phase‐change systems, demonstrating that a synapse between two neurons can be mimicked utilizing a single phase‐change system, and potentiation and depression can be generated via crystallization and amorphization of GeSbTe. f) The experiment demonstrates the use of phase‐change systems to mimic the spike‐timing dependent plasticity rule, where conduction depicts synaptic weight. e,f,) Adapted with permission.^[^ ^392^ ^]^ Copyright 2012, American Chemical Society.

To achieve analog computing in MM systems, gradual conductive switching is the key idea utilized (**Figure** **7**a). For instance, the summation of two addends or numbers, *x, y,* is implemented in a PCM layer, to facilitate an arithmetic addition (Figure 7b).^[^ ^381^, ^382^ ^]^ The high‐conductance state, which is associated with a partially crystallized state attained upon the application of *N* stimuli, and the low‐conductance state, which represents a fully amorphous PCM layer after a reset operation, are utilized to describe the upper bound and the lower bound of the output conductance. A specified number of set stimuli concomitant with both *x* and *y* are administered (the total number of crystallization pulses is below *N*), after the PCM layer is initialized to the low conductance state. By utilizing a verify and program loop, the number of remaining stimuli required to attain the high‐conductance state is examined, which results in the *N*‐complement or number, *z*, of the actual solution (the equation is expressed as *x* + *y* = *N* − *z*). Moreover, in the domain of analog computing, the idea of an accumulative MM system has been expanded to many applications, including logic operations, gradual potentiation of artificial synapses, and prime‐factor decomposition.^[^ ^383^, ^384^ ^]^ For instance, in the logic operation, in contrast to a single stimulus, Boolean‐logic computations are performed through consecutive stimuli, in which each stimulus is recorded by the PCM layer for digital summation in a logic gate. The idea of integrating analog spikes or stimuli is an extension of the summation scheme, which is a vital aspect for integrate‐and‐fire neurons in a spiking neural network (SNN) (Figure 7c).^[^ ^385^, ^386^ ^]^ The idea of an artificial synapse that obtains spike signals from various presynaptic inputs has been demonstrated. For example, in PCM layers, input spikes are combined and computed through the Kirchhoff's law of summing currents at a neuron circuitry source (Figure 7d).^[^ ^387^ ^]^ The creation of a fire condition, i.e., an output spike signal, occurs after the PCM layer accumulates incoming spikes, and reaches a high‐conductance state threshold. In contrast to the traditional charge‐integration capacitor‐type approach, in which capacitors utilize a large circuitry area that limits the number of neurons harnessed in a neuromorphic system, a principal benefit of the PCM layer is the excellent device downscaling. By utilizing a volatile RSM layer with a variable Ag filament or the threshold switching in a Mott insulator, resembling ideas of integrating neurons have been revealed.^[^ ^388^ ^]^ Additionally, RSM layers have disclosed stimulus accumulation in nanosized features, in which constant stimuli result in an increasing reset transition, owing to the steady increase in the depleted gap that separates the conduction channel.^[^ ^389^ ^]^ Analog synapses in artificial neural networks (ANNs) based on interface switching and filamentary RSM layers, together with the STT‐based MTM layer, have further harnessed the increasing conductance variation.^[^ ^390^ ^]^

For PCM layer‐based synapses and neurons, Hebbian learning’ fundamental goal is to mimic biological synapses by analyzing the strength of connections between presynaptic and postsynaptic neurons (Figure 7e).^[^ ^103^, ^391^ ^]^ According to this concept, synaptic weight between neurons increases when engaged concurrently but decreases when triggered separately. The weights between artificial neurons must be altered according to the learning principle for brain‐inspired computing applications. Spike‐timing‐dependent (STDP), a type of Hebbian learning, was accomplished using a single PCM element per synapse, resulting in artificial synaptic dynamics approximating biological synapse (Figure 7f).^[^ ^392^, ^393^ ^]^ Moreover, a succession of pre‐spike stimuli with high amplitude but narrow width is utilized in the GST‐based PCM element to induce melt quenching or crystallization, which influences the element's conductance. The distinctive responses of electronic synapses to input signals are determined by the connection between length, amplitude, and spacing of voltage stimuli. PCM‐based synapses are ultrasmall in physical size and consume a minimized amount of energy, paving the way for energy‐efficient and compact brain‐inspired computational systems. The SNNs and large‐scale neural networks based on GST synapses exhibit enhanced complex visual pattern extraction and recognition, while consuming a low energy amount.^[^ ^394^, ^395^, ^396^ ^]^ A combined hardware–software neural network design with approximately 0.2 million GST synapses attain the same accuracy as software‐based training approaches utilizing supercomputers while saving two orders of magnitude on energy.^[^ ^394^, ^397^ ^]^

Brain‐inspired computing systems are constructed by mimicking neural dynamics, i.e., neurotransmission, membrane potential maintenance, and transient dynamics. Ion channels and pumps in biological neurons maintain membrane potential, which can be adjusted by inhibitory or excitatory postsynaptic potentials. When a neuron soma is sufficiently excited or integrated, an action potential or fire signal is created that travels through the axon, with neuronal firing based on excitation history. The neural membrane potential can be emulated using phase‐change neurons with low conductance. The integrate and fire functionality can be mimicked via the crystallization of PCM layers.^[^ ^398^, ^399^ ^]^ When the conductance approaches a threshold, the neuron generates the fire signal, which is associated with a recrystallized phase. A melting voltage stimulus resets the neuron after firing. The width, amplitude, and time interval of several voltage stimuli were examined to alter the firing rate of phase‐change neurons. These neurons, when integrated with plastic synapses, have exhibited sophisticated computational tasks.^[^ ^400^, ^401^ ^]^ Moreover, GST is an important material system for examining the intrinsic stochastic character of phase‐change neurons, which is regulated by crystallization processes and may be altered by varying the chemical bond strength and amorphous topology. By doping with chemically strong but geometrically mismatched dopants, e.g., carbon or nitrogen, GST discloses enhanced stochastic character. Decreasing GeTe concentration in GST toward Sb_2_Te_3_ on the other hand results in decreased stochasticity, preserving crystallized precursor abundance while lowering structural complexity owing to Ge, and therefore boosting nucleation rate.^[^ ^402^, ^403^ ^]^ Owing to the strong Sc—Te bonds, the addition of Sc to Sb_2_Te_3_ decreases stochasticity and enhances nucleation rate.^[^ ^404^, ^405^ ^]^ This design approach alters the crystallization time from sub‐nanoseconds to sub‐milliseconds, which may modulate the firing rate of phase‐change neurons. Phase‐change synapses utilized partial set stimuli, while the reset stimulus was used in the case of phase‐change neurons.

For RSM layer‐based synapses and neurons, RSM layers imitate synaptic weight by varying charge transmission at a specified voltage, rendering the RSM layer a small hardware variant of synapses for SNNs and ANNs. The RSM layers are capable of accurately mimicking both short‐term and long‐term plasticity in spikes encoded with spatiotemporal information created by SNN neurons.^[^ ^406^, ^407^ ^]^ In RSM layers with highly mobile anions or cations, spontaneous conductance decrease occurs, imitating short‐term plasticity.^[^ ^408^, ^409^, ^410^ ^]^ An RSM synapse's synaptic weight varies in response to voltage stimuli, increasing after excitation and progressively relaxing between stimuli. This phenomenon was disclosed in an Ag_2_S RSM layer and was connected to the voltage‐induced creation and spontaneous decay of an Ag filament.^[^ ^408^, ^411^ ^]^ Modulating the migration of Ag cations within an epitaxial SiGe RSM layer results in long‐term plasticity in SNNs.^[^ ^373^, ^412^ ^]^ This generates nonvolatile linear synaptic potentiation and depression in response to varied polarity voltage stimuli. STDP is a widely utilized long‐term learning rule in SNNs that is fulfilled using RSM layers.^[^ ^413^, ^414^ ^]^ The relative timing of presynaptic and postsynaptic voltage stimuli, resulting from the ionic diffusive dynamics of biological synapses, determines the synaptic weight variation of STDP. Since traditional nonvolatile RSM layers do not exhibit these characters, STDP is emulated through waveform engineering. STDP was attained in an integrated device, viz., a nonvolatile Pt/TaO_*x*_/Ta RSM element and a volatile Pt/SiO_*x*_N_*y*_:Ag/Pt RSM element, owing to the comparable ionic dynamics in volatile RSM layers to chemical synapses.^[^ ^406^, ^415^ ^]^

For MTM layer‐based synapses, binary MTJ configurations are utilized in neuromorphic computing to store binary information in magnetic layers, with the magnetization direction pointing up or down in next‐generation memory elements. However, synaptic weights in neural networks, resembling synapses in the brain, are generally real valued. A large area and programming energy are required for traditional binary MTJ configurations to encode a single synaptic weight. The creation of analog storage entities that mimic synapses in neuromorphic networks by being plastic, nonvolatile, and allowing for alteration of stored information by input modulation is attracting interest. Magnetic layers can function as memristive layers by encoding analog data in magnetic textures.^[^ ^416^, ^417^ ^]^ Recent studies have demonstrated a spintronic memristive element using magnetic domain wall displacement in a spin valve, leading to varied conductance states.^[^ ^418^, ^419^, ^420^ ^]^ This memristive capability in MTJ configurations was experimentally disclosed in recent works.^[^ ^421^, ^422^ ^]^ Experiments have shown the utilization of magnetic skyrmions to model a spintronic memristive element,^[^ ^423^, ^424^ ^]^ whereas analog‐based character in antiferromagnetic CuMnAs spintronic elements by current‐induced modulation of the Néel vector in submicron scale domains is revealed by recent investigations.^[^ ^425^, ^426^ ^]^ Moreover, the experiments have exhibited the use of SOT switching to control a memristive element in an antiferromagnet–ferromagnet bilayer system,^[^ ^427^, ^428^ ^]^ utilizing the alterations in switching currents passing through minimized magnetic domains with various orientations and exchange bias magnitudes.^[^ ^429^, ^430^ ^]^

### 4.2 Physical Unclonable Functions and Random Number Generators

**Figure 8:**
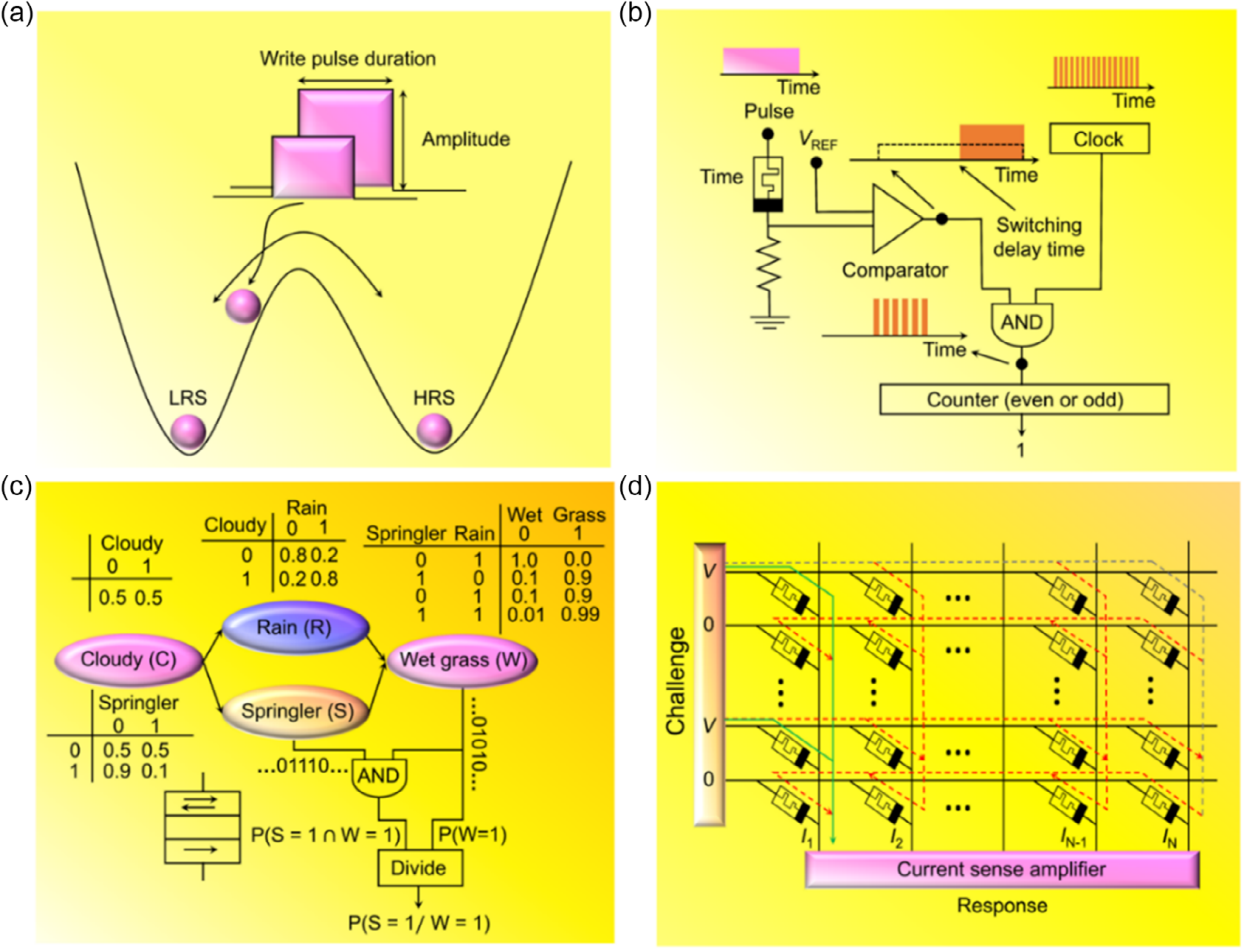
The physical unclonable functions and random number generators based on MM systems. a) Conductance switching in traditional RSM layers may be stochastic and is controlled by varying the length and voltage of programming stimuli. b) Illustration of a digital circuitry that harnesses the MM stochasticity for the creation of true random numbers. The MM unit is linked to a resistive element in a voltage divider configuration type, and a specified‐duration set stimulus is administered to the MM unit. The comparator element generates an output bit “1” owing to the set transition in the MM unit after a stochastic delay period. The counter in units of a designated clock time determines the variation between the stimulus length and the delay duration. If the variation between the stimulus length and the delay duration is an even multiple of the clock time, the output bit “0” results, whereas the output bit “1” occurs for the case when the variation between the stimulus length and the delay duration is an odd multiple of the clock time. A stochastic‐bit assemblage appears upon the application of a series of set stimuli. b) Adapted under the terms of the CC‐BY Creative Commons Attribution 4.0 International license (https://creativecommons.org/licenses/by/4.0).^[^ ^534^ ^]^ Copyright 2017, The Authors, published by Springer Nature. c) Schematic representation of a Bayesian network, wherein each node describes random variables, and every link denotes a specified dependence among nodes, determined based on transitional conditional probabilities. From a stipulated observation, these networks can be harnessed to determine the probability of concealed origins. Utilizing stochastically switching MM systems, the probability distribution required for implementing probabilistic inferences can be created. For instance, model probabilities can be encrypted within Poisson‐distributed binary bit ensembles created in an MM system. By multiplying two separate bit assemblages using an AND gate, corresponding calculations including the intersection operation can be administered. Adapted under the terms of the CC‐BY Creative Commons Attribution 4.0 International license (https://creativecommons.org/licenses/by/4.0).^[^ ^535^ ^]^ Copyright 2017, The Authors, published by Springer Nature. d) Schematic depiction of MM crossbar arrays utilized for creating physical unclonable functions (PUFs). To achieve an increased group of challenge‐response pairs (CRPs), a broad distribution of conductance values is harnessed. For instance, a challenge comprises an *N*‐bit vector administered to *N* rows in an *N* × *N* crossbar PUF. The output current from *N* columns was recorded and modified to an *N*‐bit response. 2^*N*^ is the theoretical number of CRPs. Adpated with permission.^[^ ^536^ ^]^ Copyright 2016, Institute of Electrical and Electronics Engineers.

In‐memory computing also harnesses the stochasticity related to the switching signature of MM systems (**Figure** **8**).^[^ ^5^ ^]^ For MTM layers, the programming time and the input voltage are utilized to modulate the switching probability (Figure 8a), and the conventional MTJ switching may be stochastic owing to thermal fluctuations influencing the magnetic free layer. The set transition occurs after a specified delay period upon the application of a set voltage *V*_set_ in traditional RSM layers.^[^ ^431^, ^432^ ^]^ The cycle‐to‐cycle statistical variability can be disclosed over the delay period. Additionally, this phenomenon may be exhibited in prototypical PCM layers, owing to output variations connected with the low conductance state and the threshold switching process.^[^ ^103^ ^]^ The delay period dependence for different electrical voltages renders an avenue to adjust the conductance distribution in the PCM layer. Besides, archetypical PCM layers may disclose stochasticity concomitant with the crystallization time. After a reset operation, a small disparity in the atomic arrangement of traditional amorphous layers can occur. This occurrence may lead to a fluctuation concomitant with the number of stimuli required to fully crystallize the typical amorphous layer.^[^ ^433^ ^]^

The variability in traditional RNGs is not exclusively an in‐memory computing tool, but it is vital in data security and cryptography. Random key generation is also required in a PUF, which is a one‐way function for hardware chip authentication that prevents chip counterfeiting and hacking by assuring the function cannot be cloned or recorded.^[^ ^434^, ^435^, ^436^ ^]^ Random number generation is also an important tool in probabilistic SNNs for simulating synaptic neurotransmitter release or membrane channel opening and closing.^[^ ^437^, ^438^ ^]^ Traditional approaches create seed‐dependent pseudo‐random numbers using software and hardware strategies.^[^ ^439^, ^440^ ^]^ A physical entropy source, e.g., variability or noise in memory elements, is required to construct a true random number generator (TRNG). Additionally, for many domains, e.g., deep learning, machine learning, stochastic computing, and data encryption, random number generation using the MM system is important.^[^ ^441^, ^442^ ^]^ For achieving an effective and portable TRNG, utilizing MM systems as a disorder source has attracted substantial interest. The TRNG harnesses the entropy through physical effects, including ring‐oscillator jitter, time‐dependent dielectric breakdown, and Johnson–Nyquist noise,^[^ ^443^, ^444^ ^]^ and does not involve a seed, in contrast to a conventional pseudo random‐number generator (PRNG). Stochastically switching MM systems with simple circuitries, viz., digital logic components and a comparator, are utilized to achieve the TRNG (Figure 8b).^[^ ^445^ ^]^ Different types of TRNGs have been examined through MM systems.^[^ ^446^, ^447^, ^448^ ^]^ Moreover, to achieve effective multiplication operations, stochastic number streams created by a TRNG element through MM systems have been utilized.^[^ ^449^ ^]^ For instance, by implementing an AND operation between binary random bit streams for two numbers ranging from 0 to 1, the multiplication operation of these numbers was attained successfully.^[^ ^450^ ^]^ Administering a probabilistic inference through the Bayes's rule is another emerging application (Figure 8c). For example, by harnessing a stochastically switching MTM layer, the requisite probability distribution was created as a random‐bit stream.^[^ ^451^ ^]^

The realm of security is another emerging application of MM systems. A physical scheme that statistically maps input data to outgoing output data through a secret key determined by an inherently stochastic character of a memory element is termed a PUF.^[^ ^452^ ^]^ Experiments have harnessed the intrinsic physical variation of device parameters and material process variabilities. A computational unit that returns an output response, *r* = *f*(*c*), for each input challenge *c*, represents the PUF. The inner physical signature of the PUF is denoted by the *f*. An assemblage of potential challenge‐response pairs (CRPs) describes a targeted PUF sample.^[^ ^453^ ^]^ By utilizing metastable states of cross‐coupled inverters, traditional charge‐based material systems are harnessed to achieve PUF circuitries. However, to develop a strong PUF with a large CRP ensemble, MM systems assembled in a crossbar configuration have been utilized (Figure 8d). Harnessing the large number of current sneak paths and the wide distribution of conductance values, is the main concept.^[^ ^454^ ^]^

## 5 MM‐Based In‐Sensor Computing

**Figure 9:**
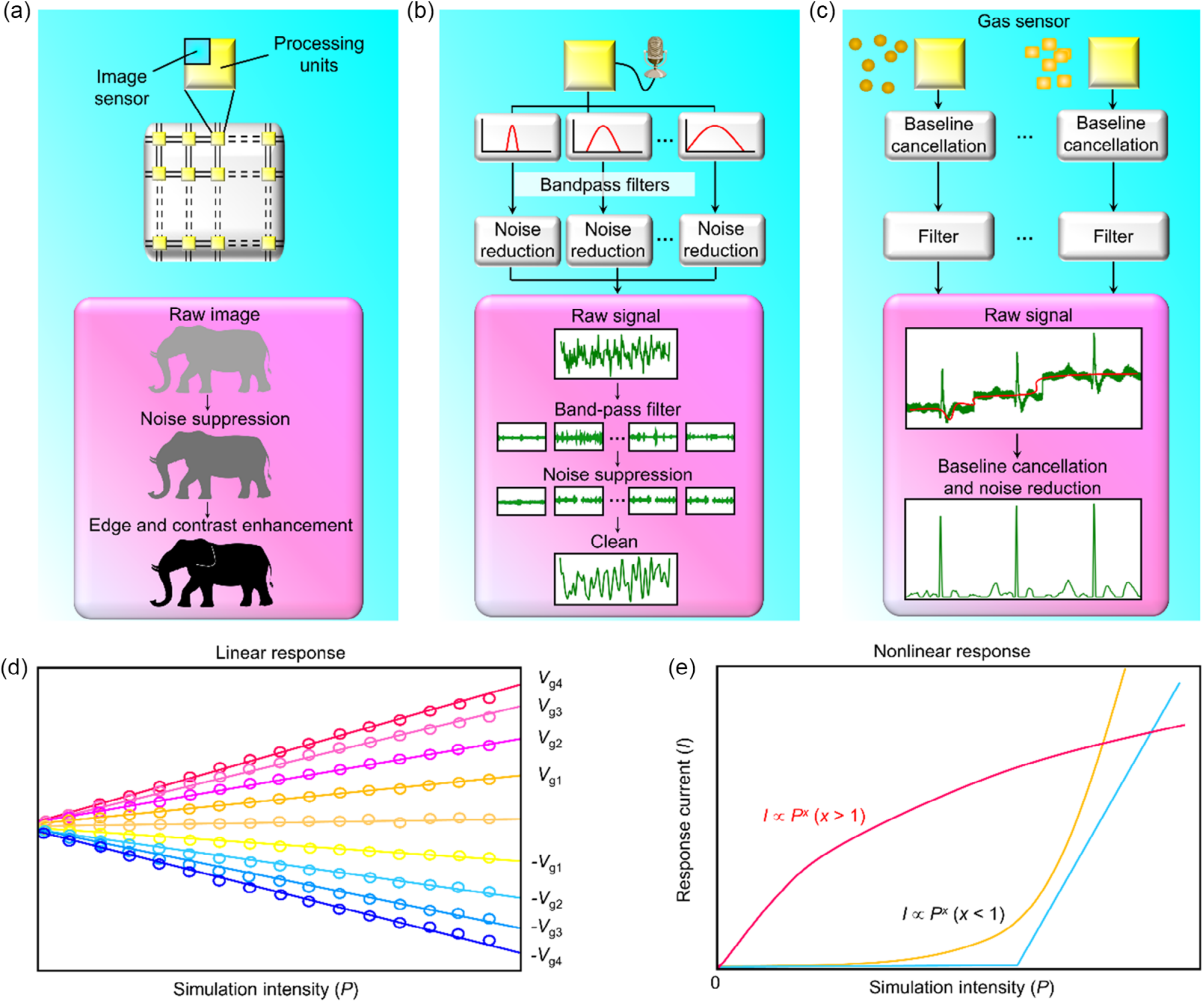
MM systems for in‐sensor computing. a) Illustration of the low‐level visualization processing methodology using edge‐removal and contrast improvement processes. The visualization processing and detection schemes are integrated in the same sensor for in‐sensor computation. b) Schematic diagram of the low‐level acoustic processing approach. To achieve clear signals for subsequent processing, the pure acoustic signal is refined utilizing band‐pass filters with a noise suppresion in every channel. c) Schematic representation of low‐level aromatic processing strategies. The distinction of gas modes in subsequent high‐level processing methodologies is influenced by the baseline difference in pure sensory information. The baseline was extracted from input signals. d,e) Various sensory response characters for in‐sensor computing. Linear response signatures enhance precision in in‐sensor computing on artificial neural networks, as shown in (d), whereas nonlinear sensor signatures, e.g., sublinear, threshold, and superlinear, generate intensity‐dependent information, enabling spatiotemporal information encoding and improving computation functions at sensory terminals, as depicted in (e). d,e) Adapted with permission.^[^ ^465^ ^]^ Copyright 2022, Springer Nature.

Varying from low‐level sensory data to high‐level abstract depiction, hybrid sensory and computing hardware with MM systems disclose a feed‐forward and hierarchical character.^[^ ^455^ ^]^ By improving elements for further processing or minimizing undesirable distortion or noise, low‐level processing schemes initially and critically obtain valuable information from a large amount of unprocessed information, which are vital processing steps in data‐intensive applications.^[^ ^456^ ^]^ The outputs of the low‐level processing strategy are still depictions of a sensory signal in this phase. The computing efficacy increases and the computational task decreases for achieving high‐level processing workloads when low‐level sensory processing methodologies, e.g., motion removal, noise minimization, element improvement, background removal, filtering, and other methodologies, are utilized.^[^ ^44^ ^]^ Implementing simple and targeted operations through a reading circuitry is facilitated by the low‐level processing strategy, which performs as the interface between other high‐level processing systems and sensing. Recent reports have demonstrated the utilization of low‐level processing approaches for aromatic, acoustic, and visualization signals prior to and upon the processing mode (**Figure** **9**). Additionally, image sensors with MM systems are built using a CMOS‐compatible procedure over a broad scale, among many sensors. The image processing scheme is a data‐intensive computational workload owing to an increased frame rate and a large pixel density. Noise minimization, as well as contrast and edge improvement, are involved in the low‐level image processing technique.^[^ ^457^ ^]^ By utilizing an arithmetic and logic‐in‐sensor system, which implements arithmetic functions and logic operations of sensory signals directly in the same sensor, circuitries at the front end of the CMOS process are minimized. Through harnessing new material systems, including the optoelectronic MM system or switch‐based material platforms, arithmetic and logic‐in‐sensor operations have been performed.^[^ ^458^, ^459^ ^]^ Noticeable feedbacks to various combinations of inputs have been disclosed by MM systems, and the MM system generates varying conductance levels.

With the emergence of the Internet of Things, the number of sensor nodes is ever increasing and is anticipated to reach ≈45 trillion by 2032.^[^ ^460^, ^461^ ^]^ The data creation, which is expected to exceed 1 million zettabytes per year, exhibits substantial time latency and energy consumption concerns. Since the large volume of data created by the sensors obscure vital information, a computing paradigm is required to effectively decrease frequent data transfer, analyze information inside sensors, and minimize redundant data. Owing to variations in manufacturing approaches, traditional sensors and processing entities are physically separated, resulting in low energy efficiency and time delays during data transfer. Recent works have demonstrated the creation of a variety of digital elements that include both sensing and computation capabilities, enabling in‐sensor computing and avoiding data transfer outside of sensors.^[^ ^462^, ^463^, ^464^ ^]^ Moreover, different compute functions may be attained using the response signatures of the sensors (Figure 9d,e).^[^ ^44^, ^465^, ^466^ ^]^ Additionally, vision sensors are an excellent example of in‐sensor computing, allowing humans to collect data from the surroundings.^[^ ^467^, ^468^ ^]^ Image sensing utilizes optoelectronic conversion to facilitate information reconstruction and reorganization. These technologies enable visual information to be computed in‐sensor. Sensors exhibiting linear and nonlinear response signatures are utilized to recognize images or improve features within a sensor array.^[^ ^455^, ^464^ ^]^ The linear response sensor was used in combination with ANNs to accomplish computation. Machine leaning methodologies are attained using summation and multiplication, with hardware implementation via optoelectronic conversion. The multiplication function $I_{\text{ph}}=R\times P$ is utilized, where $I_{\text{ph}}$ is the photocurrent, *R* is the photoresponsivity, and *P* is the power intensity of light stimulation. The gate voltage constantly adjusts photoresponsivity, mimicking synaptic weight in ANNs. By linking photosensors in series, Kirchoff's current rule was achieved. Recent studies exhibited ultrarapid image recognition in nanoseconds,^[^ ^469^, ^470^ ^]^ whereas utilizing Si technology, other studies improved on this image recognition methodology.^[^ ^464^, ^466^ ^]^ Photosensors disclose nonlinear response signatures that allow the photosensor to recreate optical inputs, allowing for different methodologies of processing visual information. Superlinear response sensors generate large photoconductance levels, whereas a small photoconductance level results in the case of weak inputs. This leads to enhanced image character and decreased background noise. The modulation of photoresponsivity across a wide range results in sublinear response abilities.^[^ ^471^, ^472^ ^]^ This approach is beneficial for in‐sensor vision modifications under various lighting settings, as well as for nociceptors that respond to inputs over a specified threshold.^[^ ^473^, ^474^, ^475^ ^]^

## 6 Outlooks, Challenges, and Opportunities

For brain‐inspired and analog computing, MM systems have been utilized to mimic biological synapses.^[^ ^392^, ^393^, ^476^, ^477^, ^478^, ^479^ ^]^ The main signatures of the biological synapse, viz., the synaptic plasticity and the synaptic efficacy, are disclosed by the MM system.^[^ ^480^ ^]^ The ability to vary the synaptic weight in accordance with a learning rule is described as the synaptic plasticity, while the synaptic efficacy represents the process of creating an output according to incoming spikes. For the synaptic‐efficacy signature, the stored weight of the synapse, viz., the nonvolatile, analog, reconfigurable conductance, is multiplied with the incoming spike. The multiplied signal of pre‐neurons, i.e., the neuron in specified layers that obtained the input spike, was added and then administered as an input signal to the post‐neuron, viz., neurons in a targeted layer that created the output spike. Emerging nonvolatile MM systems assembled in a crossbar configuration have been utilized to demonstrate the in situ synaptic plasticity and the synaptic efficacy.^[^ ^481^ ^]^ To construct large scale, dense neuromorphic processors for achieving in situ in‐memory computing, memristive crossbars are further combined in an event‐based mode using the networks‐on‐chip (NOC).

Synaptic learning with STDP rules and in situ dot‐product operations have been demonstrated in many experiments using MM systems.^[^ ^482^, ^483^, ^484^ ^]^ The RSM layer utilizes the filament generation to attain a reconfigurable and analog conductance.^[^ ^485^ ^]^ However, cycle‐to‐cycle and device‐to‐device variations may be disclosed in traditional RSM layers, which is a performance bottleneck. The PCM layer, consisting of a chalcogenide alloy sandwiched between two metal electrodes, elucidates reversible switching between the amorphous state, i.e., the low conductance state, and the crystallized state, viz., the high‐conductance state.^[^ ^486^ ^]^ Although conventional PCM layers may exhibit a resistance drift over time and a large programming current, the PCM layer discloses a short switching time, as well as a low writing voltage. Recently, a superlattice‐type PCM layer with alternating material sublayers has been demonstrated as a promising way to mitigate the resistance drift phenomenon and achieve a multibit capability in both CMOS‐compatible rigid and flexible substrates.^[^ ^6^, ^487^ ^]^ Moreover, nanoscale PCM layers based on a Ge_4_Sb_6_Te_7_ nanocomposite material have shown bidirectional gradual conductance changes with a large programming window using low energy stimuli, enabled by unique microstructural and electrothermal signatures of the epitaxial nanocomposite material.^[^ ^488^ ^]^ Two conductive states determined through whether the magnetization of two specified layers was in a parallel or antiparallel orientation were shown by MTM layers, which comprised two magnets separated by a spacer.^[^ ^489^ ^]^ The MTM layer reveals a quick reversal time, low programming energy, and high writing endurance. However, the ratio of on‐state conductance levels to the off‐state conductance level may be small in prototypical MTM layers. Besides, various challenges exist, although the synaptic learning and in situ operations provide promising outlooks for achieving a distributed, beyond von Neumann, large‐scale computing in MM systems. The estimated character of traditional computations is susceptive to errors that decrease the application accuracy, along with the computing efficiency, owing to process‐type, cycle‐to‐cycle, and device‐to‐device variations. Additionally, the existence of sensing resistance, source resistance of driving circuitry, line resistance, and current‐sneak path influence the strength of conventional crossbar‐array operations. The complexity of developing strong computations using traditional synaptic elements is augmented by small bit precision, utilization of analog‐to‐digital converters, nonidealities of selector elements, and low energy efficiency. Inaccurate programming operations that require the repeated and costly program‐and‐verify methodology result from the intrinsic stochastic signature of the conventional synaptic element.

For PUFs and RNGs, in which MM systems are not utilized to decrease data transfers, a general advantage results for the TRNG accelerator compared to that for a traditional realization. Since most commercial systems utilize the CMOS technology, the majority of conventional PUF designs center on harnessing process variations inherent to CMOS technologies. In MM systems, a substantial progress in nanosized elements has been achieved.^[^ ^42^, ^490^, ^491^ ^]^ To develop memory units based on storage signatures, viz., magnetism and memresistance, MM systems are promising candidates. Moreover, when elements are scaled down to the nanometer lengthscale, large fabrication variation phenomena are an obstacle for archetypical MM systems, which is undesirable for memory‐type applications since the reading margin decreases. However, when developing PUFs, this intrinsic entropy is utilized to a positive benefit.^[^ ^492^, ^493^ ^]^ Highly reliable PUFs are achieved by harnessing conductance variations in the On state, i.e., logic “1” and the Off state, viz., logic “0”, which disclose a bimodal‐conductance distribution.^[^ ^494^ ^]^ Moreover, MM systems are compatible with the CMOS fabrication procedure with 3D integration competencies and are simple to construct. These signatures enable various device layers to be combined in a stack to alleviate different side‐channel attacks. The tamper resistance also increases when the PUF is inserted in the middle device layer.^[^ ^495^ ^]^ Furthermore, the simple nanosized‐crossbar design renders a low‐cost PUF methodology and enables ultrahigh density data storage.^[^ ^496^ ^]^ Furthermore, recent opportunities for achieving unique PUF designs are furnished by distinct characters of the MM system. For example, experiments have disclosed a PUF that utilizes the bidirectional signature of RSM layers, as well as cycle‐to‐cycle variations in programming the RSM layer.^[^ ^42^ ^]^ Furthermore, MM systems are resistant to photon emission attacks. For instance, photons are not emitted in the trap‐assisted tunneling conduction process of the RSM layer.^[^ ^497^ ^]^

For in‐sensor computing, MM systems are required to cooperate with algorithms. Owing to inherently digital signatures, traditional silicon CMOS systems do not exhibit high efficacy for neural‐network algorithms. The leading contenders for achieving in‐memory computing in intelligent workloads and hardware realization of ANNs are next‐generation neuromorphic computing material platforms, including the MM system with high stackability, low programming energy, short switching time, varied analog conductance states, high linearity and symmetry, adaptable plasticity, and minimized footprint.^[^ ^498^, ^499^, ^500^ ^]^ Furthermore, by combining sensing operations in MM systems, the computing sensor architecture can be harnessed for in‐memory computing.

At the circuit level, MM computing platform performance is influenced by aspects besides MM systems, e.g., periphery circuitry, parasitic resistance and capacitance of crossbars, signal representation, and crossbar interconnections. For instance, computational accuracy, signal representation, and throughput influence the complexity of peripheral circuitry. Various signal‐generation and acquisition systems have been documented, with substantial differences in chip energy and footprint. Transimpedance amplifiers and analog‐to‐digital converters can sense analog currents,^[^ ^501^, ^502^, ^503^ ^]^ whereas digital‐to‐analog converters can generate analogue voltage inputs. The energy efficiency of ≈50 TOPS W^−1^ with MM‐based arrays has been demonstrated using on‐chip current‐sense amplifiers and digital inputs.^[^ ^504^, ^505^, ^506^ ^]^ Moreover, wire resistance and parasitic capacitance may constrain the size of a single MM crossbar, influencing vector‐matrix multiplication accuracy and limiting the upper bound of programming time. Circuit‐level mitigation approaches, viz., MM systems with low working conductance and numerous small crossbars with reconfigurable interconnections, can facilitate minimize these difficulties. This, however, generates a large resistance‐capacitance (RC) constant. Additionally, analog voltages are the most frequently used signal representation in ANNs and analogue computers. Signals can also be encoded using the number or temporal length of identical voltage stimuli. This methodology requires extended time but may minimize the use of various bias levels within a small voltage range. It also avoids the requirement for metallic or ohmic RSM layer transport, which is typically present in the high‐conductance state, allowing low‐conductance‐state MM systems to achieve high energy efficiency. Furthermore, various MM crossbars may be required in real‐world applications, and reconfigurable interconnections between them may allow for flexible adjustment to different MM network topologies.^[^ ^507^, ^508^ ^]^

## 7 Conclusion

A market for effective and high‐performance training and inference systems, both on the edge and in the cloud, has been generated by the rapid growth of AI, e.g., deep neural networks. A vital role in defining the future of computing is represented by mobile devices, which are impeded by energy limitations. The leveling of the cost per transistor as the transistor size becomes smaller is another reason. Various device manufacturers could be motivated to maintain previous technology nodes or feature sizes but enhance the device performance with high‐efficacy computing elements including a computational memory. The integration of the MM system with a variety of front‐end CMOS technologies is facilitated, since most MM systems are compatible with the BEOL integration. In‐memory computing based on the MM system is expected to have a substantial impact on enhancing the area and energy efficiency, along with the latency. This could pave a way for the next generation of non‐von Neumann computing in a favorable market setting.

## Conflict of Interest

The authors declare no conflict of interest.
